# Supplementary material for: A Refined Open State of the Glycine Receptor Obtained via Molecular Dynamics Simulations
Source: Structure. 2020 Jan 7;28(1):130–139.e2. doi: 10.1016/j.str.2019.10.019 (PMC6945115; doi:10.1016/j.str.2019.10.019)
Supplement: Document S1. Figures S1–S8 [file mmc1.pdf]

Structure, Volume *28*

Supplemental Information

A Refined Open State of the Glycine Receptor Obtained via Molecular Dynamics Simulations

Marc A. Dämgen and Philip C. Biggin

Supplementary Information

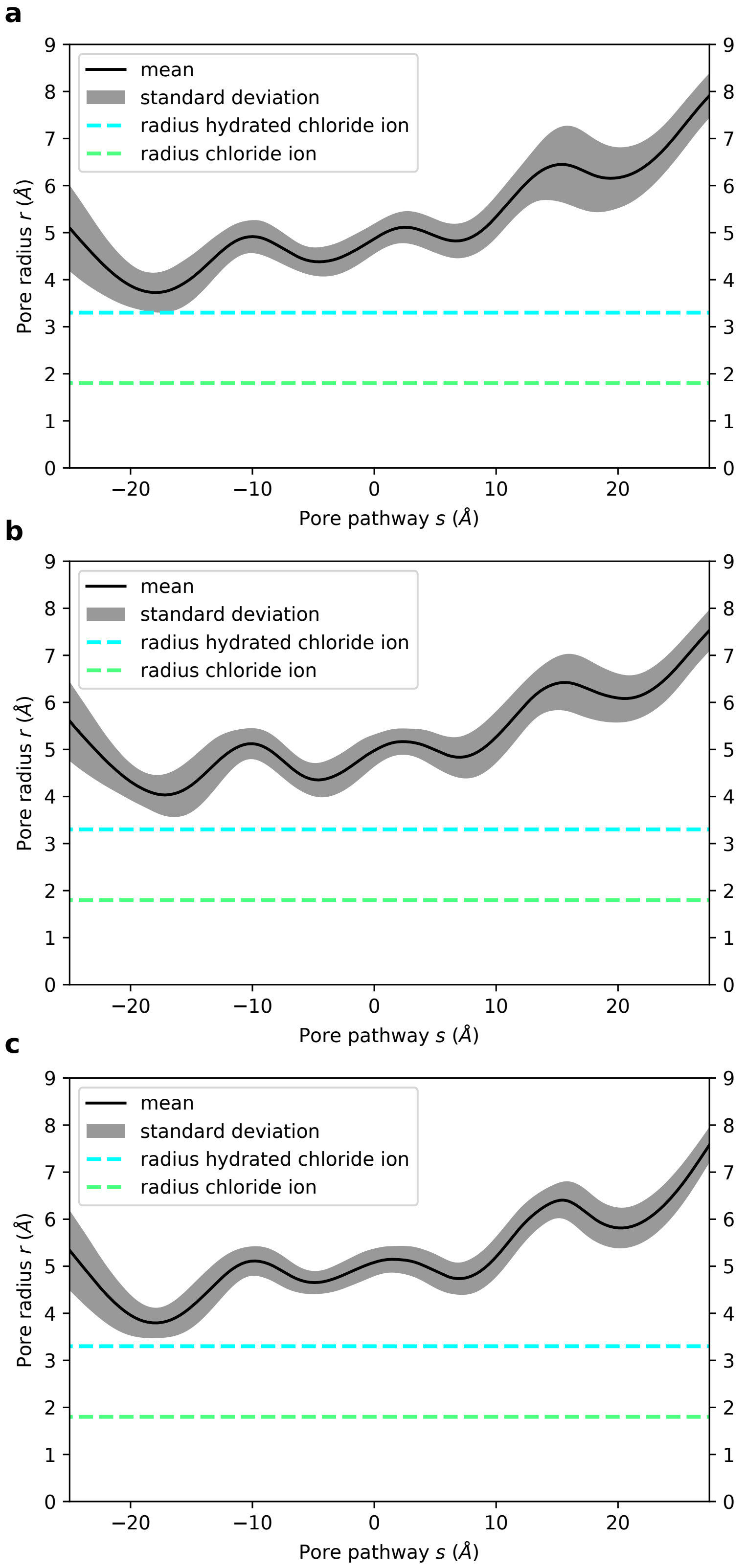

Supplementary Figure 1. Related to Figure 1. Pore profiles from three independent simulations with a stable open state. Time-averaged pore radius profiles of the transmembrane domain obtained from three independent simulations (of 300 ns length each) initiated with different velocities from the system obtained after 150 ns equilibration with pore restraints. No restraints were applied to keep the pore open. The radii of a dehydrated and a hydrated chloride ion are indicated by dashed green and cyan lines, respectively. The pore is positioned in this and subsequent figures such that the L9’ ring is located at s = 0. In all three repeats, the structure from the simulation is physically open and theoretically allows permeation of hydrated chloride ions.

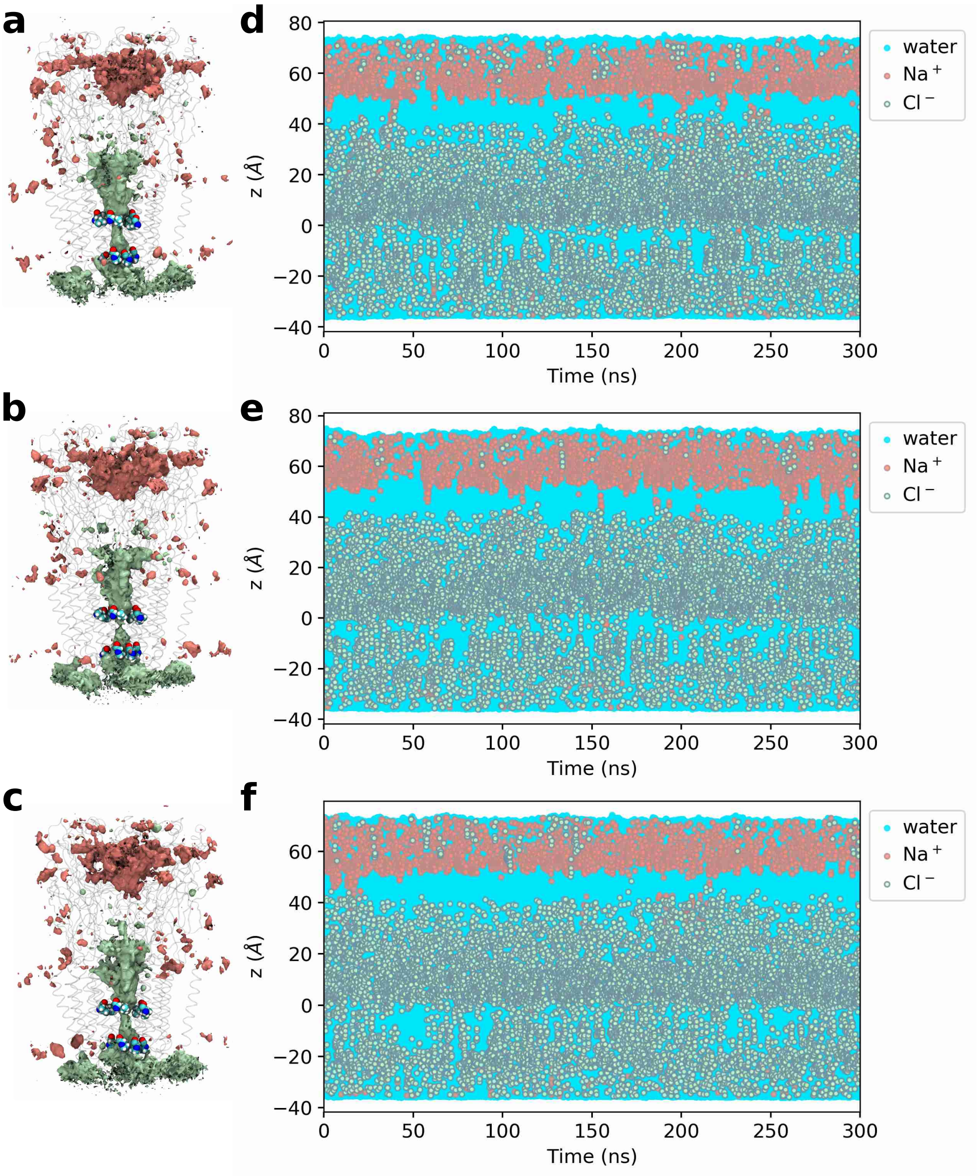

Supplementary Figure 2. Related to Figure 2. Ion densities and trajectories of water and ions within 5 Å of the channel axis for simulations with a stable open state (*150mM NaCl with no membrane potential*). (a-c) Densities of chloride (green) and sodium (red) ions over 300 ns. The isosurfaces represent a density value of 0.5 particles/nm^3. Transparent ribbons indicate the receptor backbone. The L9’ ring and P-2’ ring residues are shown in van der Waals representation. The densities prove chloride occupancy in the transmembrane pore and, moreover, show selectivity for chloride over sodium in this region. (d-f) Trajectories of water, chloride and sodium ion z-coordinates within 5 Å of the channel axis inside the pore over 300 ns (represented by blue, green and red circles, respectively). The 0 point of the z-axis is positioned at the L9’ ring and the receptor structures in the left panels are aligned and scaled correspondingly to allow for spatial orientation along the z-axis. The whole channel pore is wetted throughout the simulations (dewetted regions would appear as white stretches). While chloride frequently penetrates the transmembrane pore, sodium only does so very rarely, again demonstrating the chloride selectivity for this channel.

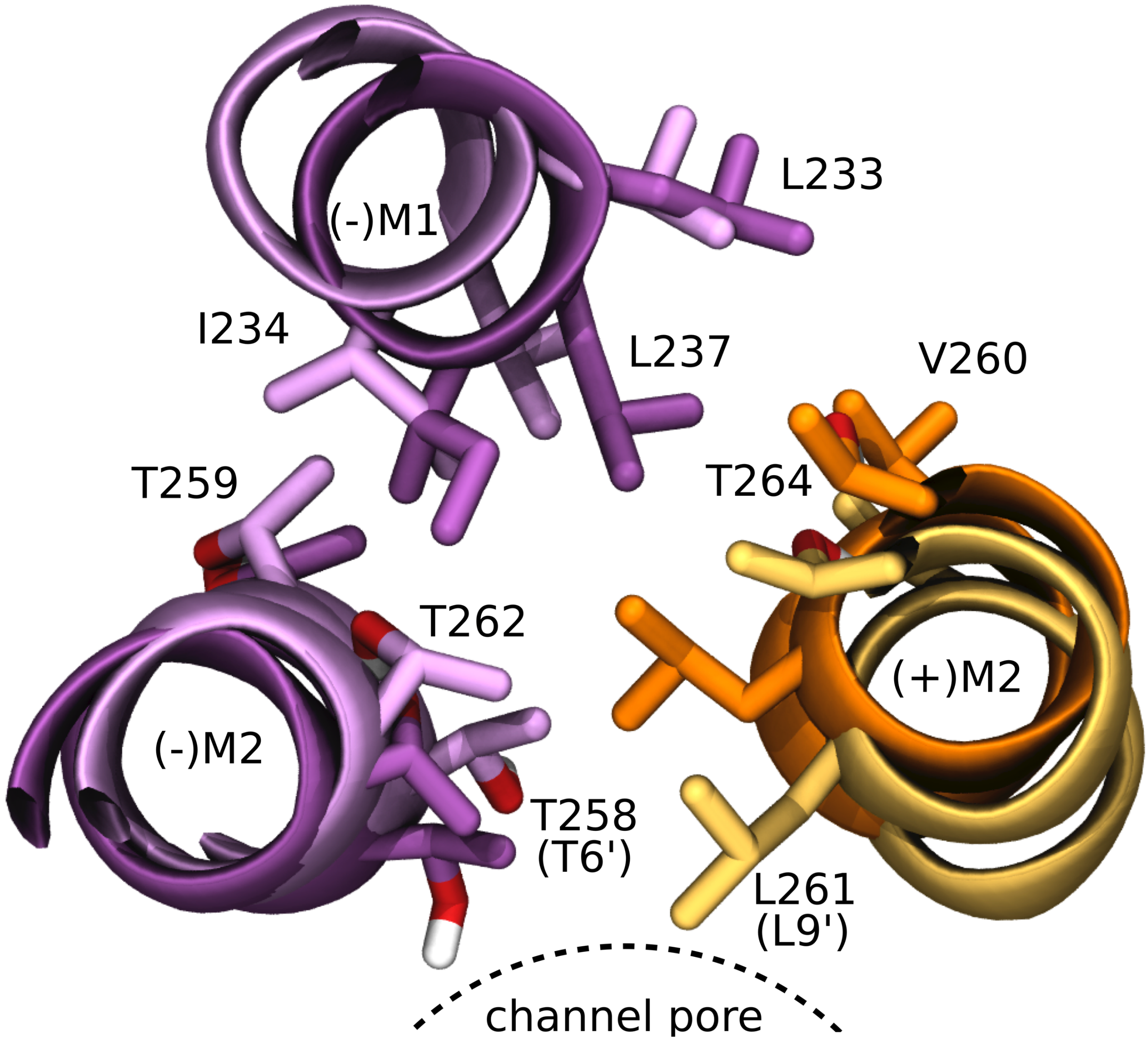

Supplementary Figure 3. Related to Figure 3. Details of the hydrophobic pore and orientation of T6'. Horizontal slice viewed from the extracellular side with the light colours representing the structure at the beginning of the equilibration with pore restraints and dark colours the structure at the end of 150 ns equilibration with pore restraints. The principal subunit is coloured in orange, the complementary subunit in purple. Key residues that form the hydrophobic pocket are indicated. Note also the position of T6' which rotates slightly towards the central pore with its hydroxyl group facing towards the channel, thus facilitating channel hydration.

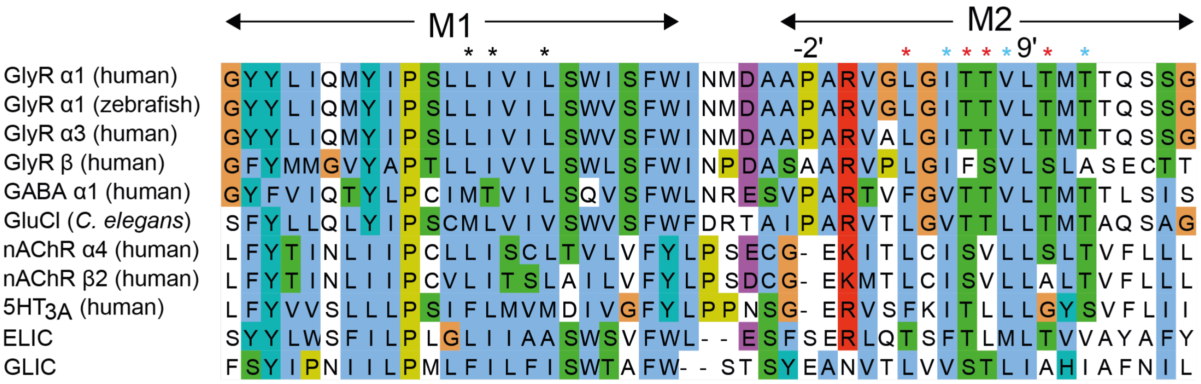

Supplementary Figure 4. Related to Figure 3. Multiple sequence alignment of pLGIC representatives. The conservation of the leucine at L9’ is well described. Less well appreciated is the functional conservation of the residues that form the hydrophobic pocket for the leucine in the open state formed by I257, V260 and T264 (I5’, V8’ and T12’ respectively and shown as cyan asterisks) belonging to the M2 helix of the principal (+) subunit, L255, T258, T259, T262 (L3’, T6’, T7’ and T10’ respectively and shown as red asterisks) belonging to the M2 helix of the complementary (-) subunit as well as L233, I234 and L237 of the M1 helix of the complementary (-) subunit, shown as black asterisks.

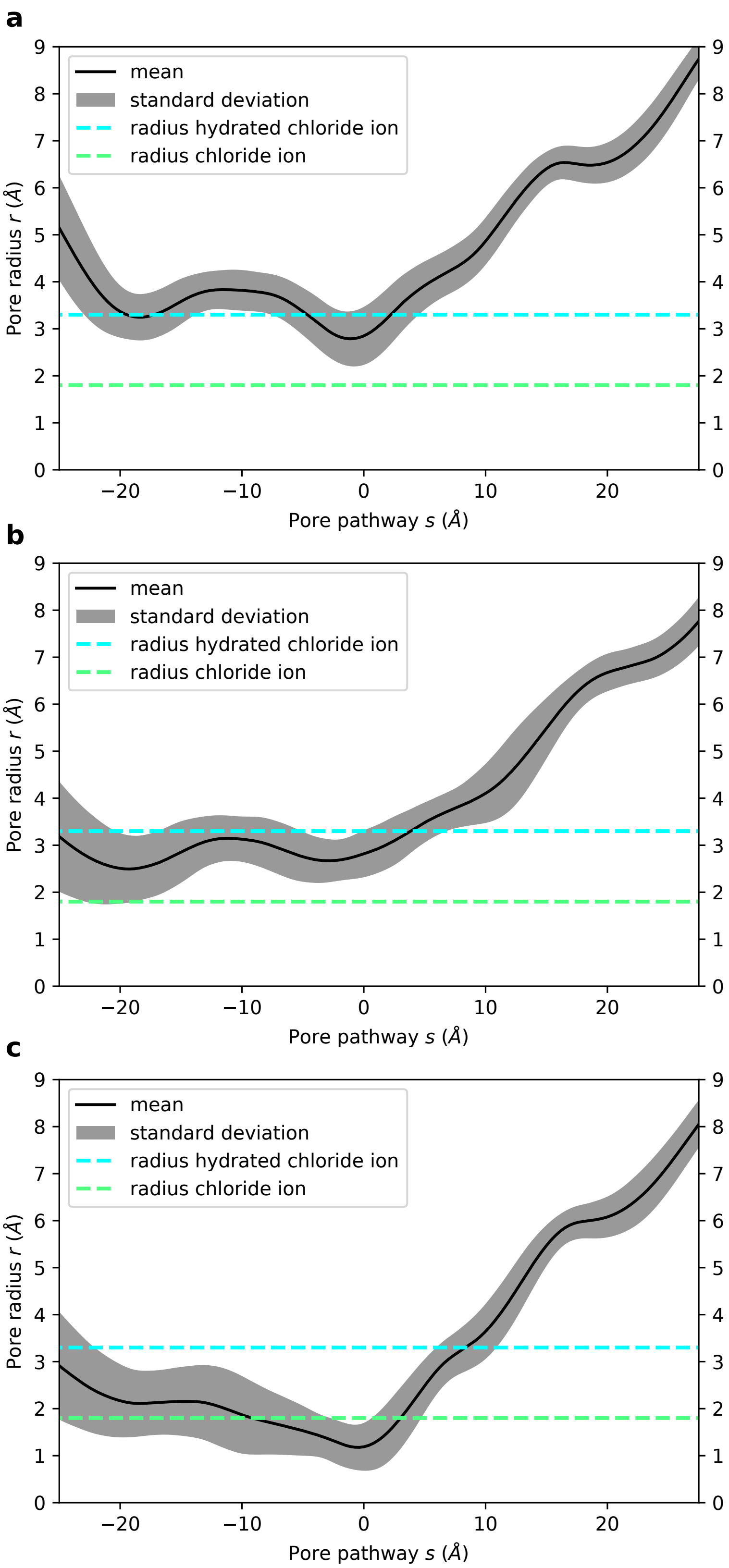

Supplementary Figure 5. Related to Figure 5. Pore profiles from three independent simulations with a collapsed state. Time-averaged pore radius profiles of the transmembrane domain obtained from three independent simulations (of 300 ns length each) initiated with different velocities from the system obtained immediately after the short initial equilibration before the structure is relaxed via the application of minimally invasive pore restraints. The radii of a dehydrated and a hydrated chloride ion are indicated by dashed green and cyan lines, respectively. The L9’ and P-2’ rings are the main

constriction points. If at all, only partially dehydrated chloride ions could theoretically permeate through the pore.

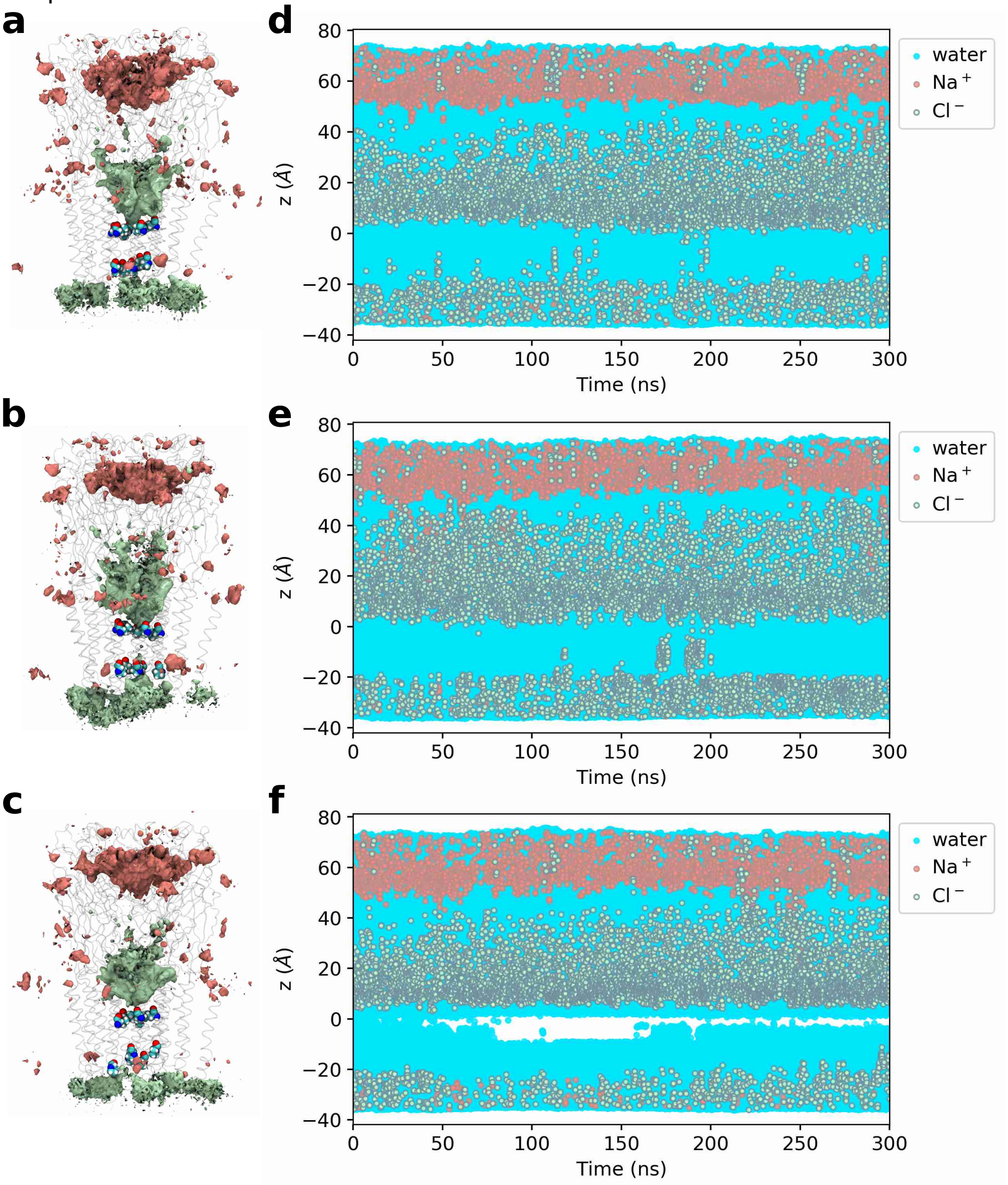

Supplementary Figure 6. Related to Figure 5. Ion densities and trajectories of water and ions within 5 Å of channel axis for simulations with a collapsed state (*150mM NaCl with no membrane potential*). (a-c) Densities of chloride (green) and sodium (red) ions over 300 ns production run. The isosurfaces shown represent a density value of 0.5 particles/nm^3. Transparent ribbons indicate the receptor backbone, The L9' ring and P-2' ring residues are shown in van der Waals representation. The densities show chloride occupancy in the transmembrane pore and moreover, show selectivity for chloride over sodium in this region. (d-f) Trajectories of water as well as chloride and sodium ion z-coordinates within 5 Å of the channel axis inside the pore over 300 ns (represented by blue, green and red circles, respectively). The 0 point of the z-axis is positioned at the L9' ring and the receptor structures in the left panels are aligned and scaled correspondingly to allow for spatial orientation along the z-axis. The white stretches in (c) demonstrate local dewetting of the pore. Chloride ions access the transmembrane pore much less frequently than in the stable open state simulations.

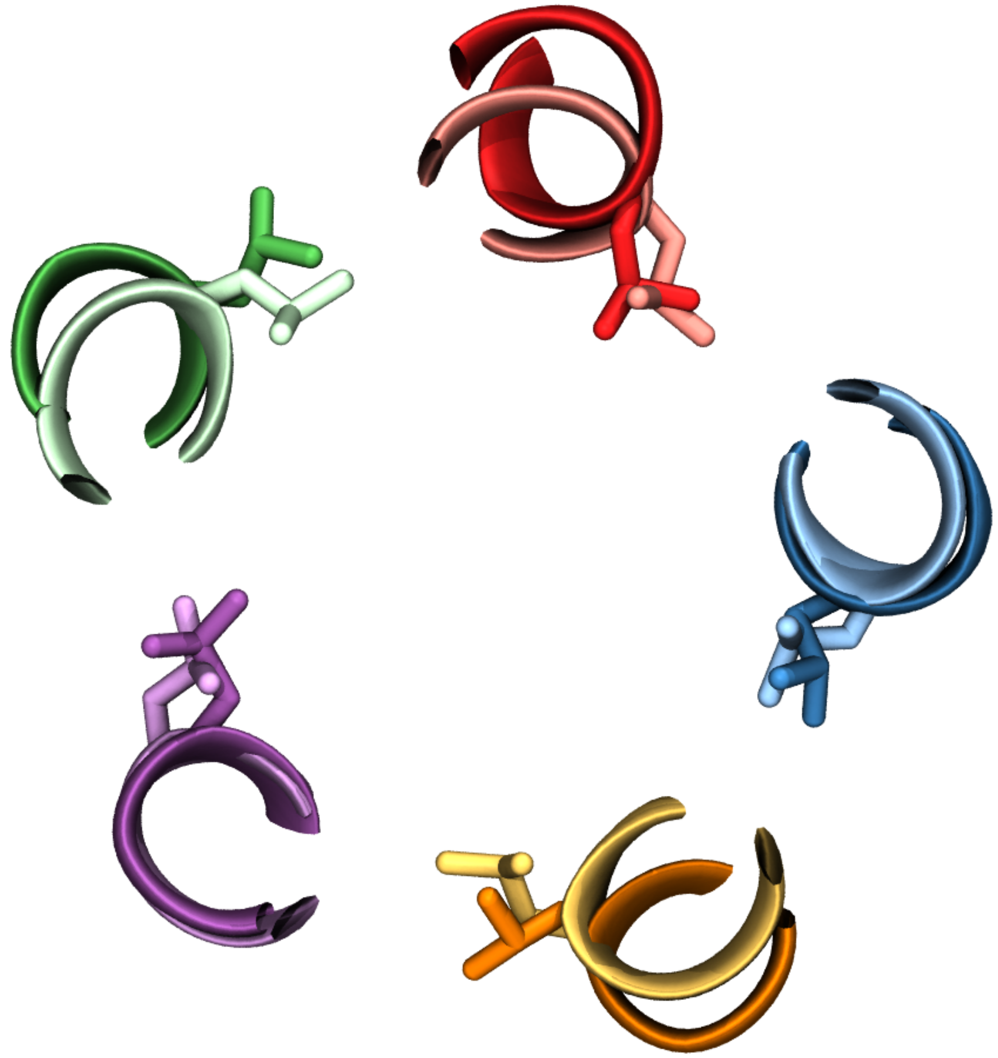

Supplementary Figure 7. Related to Figure 3. Comparison of the leucine gate between the open state glycine and the $5HT_3$ receptor. The open state model (dark colours) was fitted to the Cα atoms of L9' of the $5HT_3$ structure (light colours and PDB code: 6HIN). Shown is a top-down view of a horizontal slice at the L9' position and highlights the similarity in position of the leucines at this position in an open state.

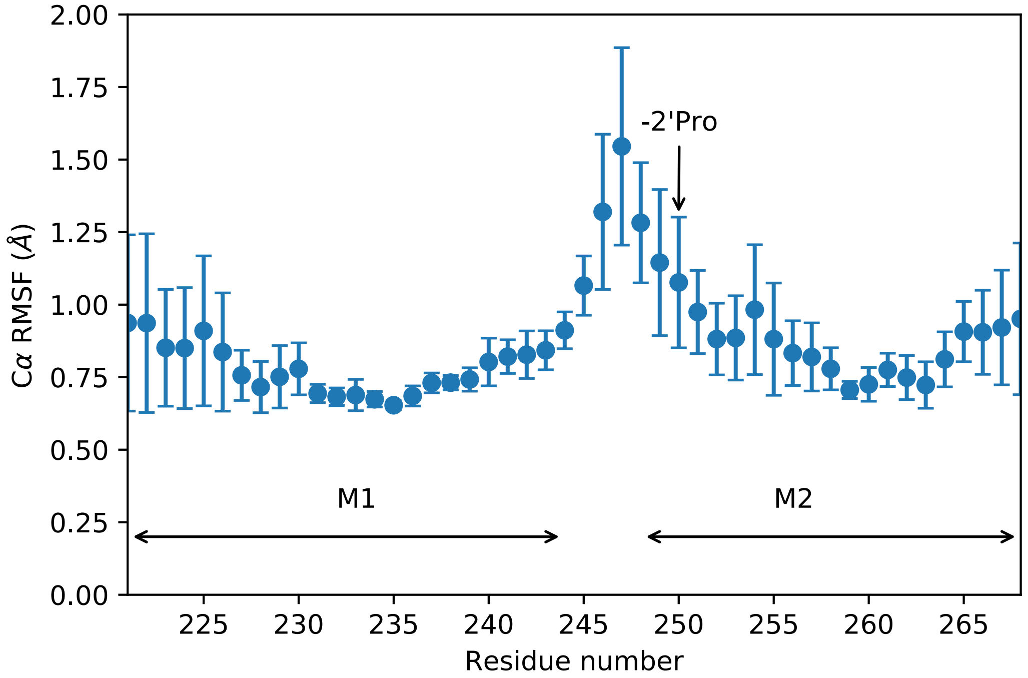

Supplementary Figure 8. Related to Figure 6. Root mean square fluctuation (RMSF) of Cα atoms of M1 and M2 helices. RMSF of Cα atoms of the M1 and M2 helices averaged over all five subunits with standard deviation as error bars. The values corresponding to the region near the intracellular pore opening of the pore lining M2 helix (where the P-2' gate is located) are very high compared to typical values for alpha-helical Cα atoms of around 0.7 Å. This indicates a high conformational flexibility that is accessible at a physiological temperature of 37 °C and can explain the poor density in the cryo-EM map in this region.
