# Supplementary material for: A Refined Open State of the Glycine Receptor Obtained via Molecular Dynamics Simulations
Source: Structure. 2020 Jan 7;28(1):130–139.e2. doi: 10.1016/j.str.2019.10.019 (PMC6945115; doi:10.1016/j.str.2019.10.019)
Supplement: Document S2. Article plus Supplemental Information [file mmc5.pdf]

Theory

Structure

A Refined Open State of the Glycine Receptor Obtained via Molecular Dynamics Simulations

Graphical Abstract

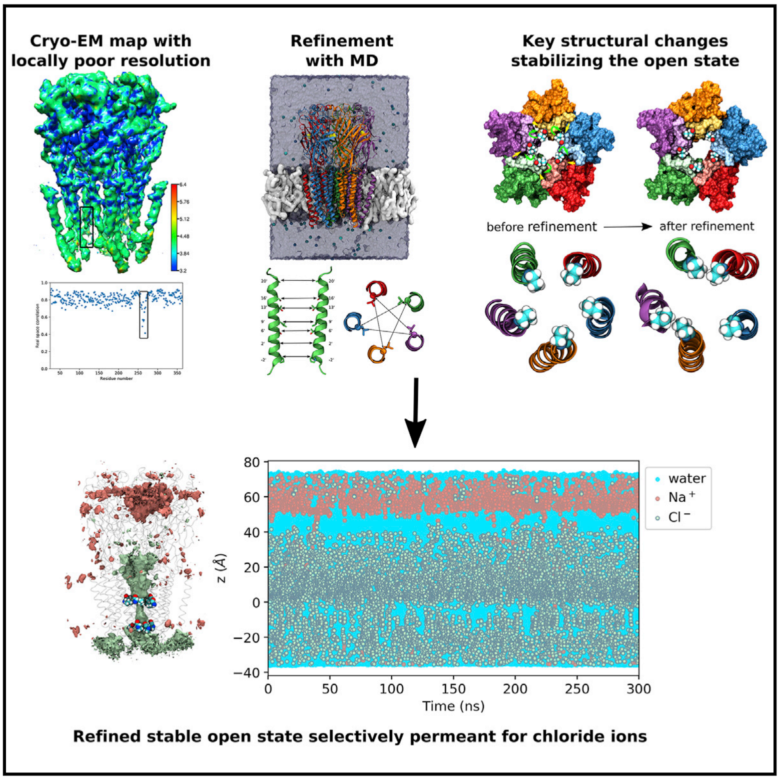

Highlights

- MD is used to refine problematic regions of the open state of the glycine receptor
- We functionally annotate it as open and selective for chloride ions
- The open state is stabilized by the 9′ residues entering conserved hydrophobic pockets
- The protocol can be more broadly applied to all members of the Cys-loop family

Authors

Marc A. Dämgen, Philip C. Biggin

Correspondence

philip.biggin@bioch.ox.ac.uk

In Brief

Dämgen and Biggin use a molecular dynamics protocol to refine problematic regions of the open state cryo-EM structure of the glycine receptor. They demonstrate that a stable open state is achieved when the leucine gate residues at the 9′ position move into well-conserved hydrophobic pockets at the interface between subunits.

Check for updates

Dämgen & Biggin, 2020, Structure *28*, 130–139
January 7, 2020 © 2019 The Authors. Published by Elsevier Ltd.
https://doi.org/10.1016/j.str.2019.10.019

CellPress

A Refined Open State of the Glycine Receptor Obtained via Molecular Dynamics Simulations

Marc A. Dämgen[1] and Philip C. Biggin[1,2,*]
[1]Structural Bioinformatics and Computational Biochemistry, Department of Biochemistry, University of Oxford, South Parks Road, Oxford OX1 3QU, UK
[2]Lead Contact
*Correspondence: philip.biggin@bioch.ox.ac.uk
https://doi.org/10.1016/j.str.2019.10.019

SUMMARY

Pentameric ligand-gated ion channels are key players in mediating fast neurotransmission. Glycine receptors are chloride-selective members of this receptor family that mediate inhibitory synaptic transmission and are implicated in neurological disorders including autism and hyperekplexia. They have been structurally characterized by both X-ray crystallography and cryoelectron microscopy (cryo-EM) studies, with the latter giving rise to what was proposed as a possible open state. However, recent work has questioned the physiological relevance of this open state structure, since it rapidly collapses in molecular dynamics simulations. Here, we show that the collapse can be avoided by a careful equilibration protocol that reconciles the more problematic regions of the original density map and gives a stable open state that shows frequent selective chloride permeation. The protocol developed in this work provides a means to refine open-like structures of the whole pentameric ligand-gated ion channel superfamily and reconciles the previous issues with the cryo-EM structure.

INTRODUCTION

The superfamily of pentameric ligand-gated ion channels (pLGICs) are key players in the communication between nerve cells. Situated in the postsynaptic membrane they mediate some, but not all, fast synaptic transmission. Therefore, they are fundamental for cognitive processes and are involved in a range of neurological disorders such as Parkinson's or Alzheimer's diseases which makes them important drug targets (Jensen et al., 2005; Gotti et al., 2006; Van Arnam and Dougherty, 2014). An atomistically detailed understanding of the functional states of these receptors would be extremely valuable for structure-based drug design.

In the resting state, the ion-conducting pore within the transmembrane domain (TMD) is closed. Upon the binding of neurotransmitters to their extracellular domain, the receptors undergo conformational changes that lead to the opening of the central pore (Nemecz et al., 2016), thereby allowing ions to pass through, leading to a change in the membrane potential and thus translating the chemical neurotransmitter signal into an electrical nerve signal. From this ion-conducting active state, they then undergo a conformational change to a non-conducting desensitized state with the neurotransmitter still bound (Katz and Thesleff, 1957). All members of the pLGIC superfamily share a very similar overall architecture (Cecchini and Changeux, 2015; Nys et al., 2013) with cation-selective channels acting as excitatory and anion-selective channels as inhibitory receptors.

The glycine receptor (GlyR) is an inhibitory member of this superfamily that is selective for chloride ions and the endogenous agonist is the amino acid glycine. Glycinergic inhibition is localized primarily in the brain stem and spinal cord where it is essential for motor coordination and pain processing (Burgos et al., 2016; Lynch, 2004, 2009). Consequently, GlyRs are a promising drug target for spasticity, inflammatory pain, and hyperekplexia (startle disease) (Lynch and Callister, 2006; Bode and Lynch, 2014; Lynch et al., 2017). GlyRs have been the focus of both functional (Lape et al., 2008, 2012; Safar et al., 2017) and structural studies and indeed various structures of the GlyRs with agonist, antagonist, and modulators bound have been resolved with cryoelectron microscopy (cryo-EM) (Du et al., 2015) as well as X-ray crystallography (Huang et al., 2015, 2017a, 2017b) and have been annotated to closed, open, and desensitized states.

From these structures and those of other members of the superfamily alongside a large amount of mutagenesis work (see Thompson et al. [2010] and references therein), it became apparent that there are two main constriction points in the channel pore, namely a ring of five hydrophobic residues (usually leucine) at the 9′ position of the pore lining M2 helices (which we hereafter refer to as the L9′ gate) and, in the case of the GlyR, a ring of five proline residues at the -2′ position. The so-called L9′ gate, located in the middle of the TMD closes the channel in the antagonist (strychnine) bound structures (PDB: 5CFB and 3JAD). The P-2′ gate is near the intracellular mouth of the channel pore and gates the channel in the desensitized-like structures (PDB: 5TIN, 5TIO, 5VDH, 5VDI, and 3JAF). Among these structures, the glycine bound cryo-EM structure PDB: 3JAE is of particular interest due to its unusually wide-open pore conformation relative to other open-like structures of the pLGIC superfamily, particularly at the -2′ position. The structure PDB: 4HFI (Sauguet et al., 2013) of the proton-activated bacterial *Gloeobacter violaceus* (GLIC) at pH 4 and the PDB: 3RIF structure (Hibbs and Gouaux, 2011) of the invertebrate glutamate-gated chloride channel (GluCl) from *Caenorhabditis elegans* were classified as open-like pLGIC structures. The distance from the pore center to the backbone Cα at the -2′ position is

 © 2019 The Authors. Published by Elsevier Ltd.
This is an open access article under the CC BY license (http://creativecommons.org/licenses/by/4.0/).

CellPress

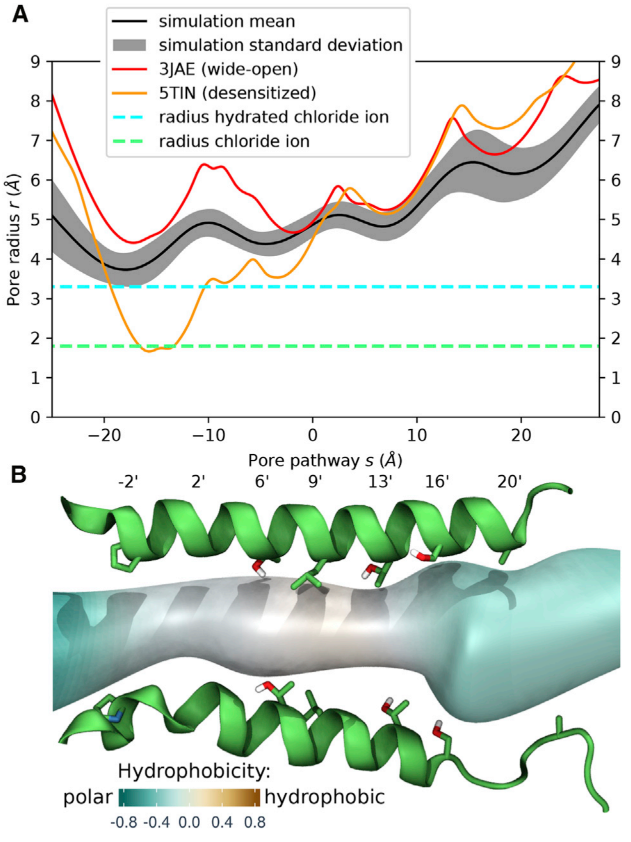

Figure 1. Pore Profile of GlyR Structures

(A) A typical pore radius profile obtained from a production run (mean, black; SD, gray) alongside the profile of the cryo-EM wide-open structure PDB: 3JAE (red) as well as the desensitized crystal structure with currently highest-resolution PDB: 5TIN (orange). The radii of a dehydrated and a hydrated chloride ion are indicated by dashed green and cyan lines, respectively. The pore is positioned in this and subsequent figures such that the L9′ ring is located at s = 0. The GlyR structure from the simulation is physically open and theoretically allows permeation of hydrated chloride ions.

(B) Corresponding pore region of a representative structure from the simulation (minimal Cα root-mean-square deviation to the average structure). M2 helices with pore lining residues (only two non-adjacent subunits shown) together with the pore volume colored by the hydrophobicity of the surrounding based on the Wimley-White hydrophobicity scale rescaled from −1 (very hydrophilic) to 1 (very hydrophobic).

about 2 Å smaller for these two structures than for the wide-open PDB: 3JAE structure (Gonzalez-Gutierrez et al., 2017).

Molecular dynamics (MD) simulations have provided useful insight into the functional annotation of states to ion channel structures, which is difficult to infer from structural information alone. An important concept in this context is hydrophobic gating (Beckstein et al., 2001; Beckstein and Sansom, 2006; Aryal et al., 2015). Hydrophobic gating occurs in narrow hydrophobic pores where the energetically favored expulsion of water prohibits ion permeation. This means that a pore whose dimensions are theoretically large enough to allow for ion permeation is not necessarily ion-permeant due to hydrophobic effects. Pore hydration has been suggested as a proxy for ion permeability (Trick et al., 2016) and used to annotate ion channels as functionally open or closed.

Gonzalez-Gutierrez et al. (2017) and Trick et al. (2016) independently annotated the wide-open PDB: 3JAE GlyR structure as open. Furthermore, Gonzalez-Gutierrez et al. found (via simulations employing a reduced model with restraints applied to backbone atoms) the PDB: 3JAE wide-open pore to have a four times higher single-channel conductance than the experimental value (although it should be noted that the simulations were performed at 37°C without the extracellular and intracellular domains present, while experiments were conducted at room temperature with the full receptor). Moreover, blocking the channel pore with picrotoxin in their simulations still resulted in ion conduction, whereas no currents were observed experimentally. This substantiates the notion that the wide-open PDB: 3JAE atomic model has an overly dilated pore. In these simulations, artificial restraints were applied to the protein backbone, such that it cannot move freely and stays very close to the original cryo-EM model. Interestingly, if no such restraints are applied, the structure undergoes a so-called hydrophobic collapse to a distinct state with a significantly smaller pore radius (Cerdan et al., 2018). This behavior has also been observed in simulations of other open (but not "wide-open") structures of the pLGIC family (Cheng and Coalson, 2012; Calimet et al., 2013; Yoluk et al., 2015). They all have in common that the hydrophobic collapse is initiated via the non-polar interaction of the 9′ gate residues.

We hypothesized that the wide-open atomic model PDB: 3JAE that has been fitted into the cryo-EM density map may not correspond to an energetically stable representation of the open state under physiological conditions. We took the view that other models that agree with the cryo-EM density map may exist and these may be conformationally stable. To find alternative open state structures, we developed a careful equilibration protocol where we give the protein maximum freedom to explore other open state configurations while preventing the pore from collapsing. We then release all restraints on the pore and show that the structure found through this careful equilibration procedure stays open with a hydrated pore that allows selectively for frequent chloride ion permeation. The model provides alternative conformations of the leucine side chains that are not discernible in the original density map. Moreover, we provide a structural explanation as to why the structure found in our MD simulations is a stable open state, whereas the wide-open structure modeled into the cryo-EM density map undergoes a hydrophobic collapse as observed by us and many others. The results reconcile previous apparently conflicting points of view and provide a way forward to model transitions between states in the pLGIC family.

RESULTS

The MD-Refined Structure Is Physically Open, Hydrated, and Selectively Permeant to Chloride Ions

We designed a careful equilibration protocol comprised of a stepwise reduction in restraint forces, followed by a series of flat-bottomed harmonic cross-channel restraints designed to keep the pore open (see the STAR Methods). The flat bottom potential only exerts a force when the Cα atoms of pore lining residues (-2′, 2′, 6′, 9′, 13′, 16′, and 20′) of non-adjacent subunits come closer to each other than in their distance in the open state

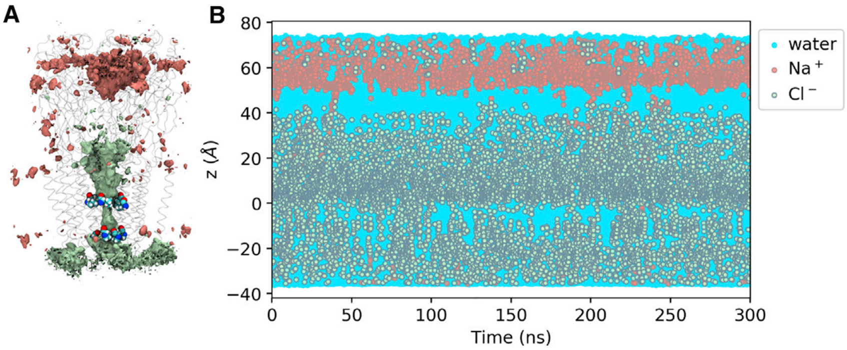

Figure 2. Ion Densities and Trajectories of Water and Ions within 5 Å of the Channel Axis in 150 mM NaCl with No Membrane Potential

(A) Densities of chloride (green) and sodium (red) ions over a 300 ns production run. The isosurfaces shown represent a density value of 0.5 particles/nm^3. Transparent ribbons indicate the receptor backbone. The L9′ ring and P-2′ ring residues are shown explicitly in van der Waals representation. The densities show chloride occupancy in the transmembrane pore and, moreover, show selectivity for chloride over sodium in this region.

(B) Trajectories of water as well as chloride and sodium ion z coordinates within 5 Å of the channel axis inside the pore over 300 ns (represented by blue, green, and red circles, respectively). The 0 point of the z axis is positioned at the L9′ ring and the receptor structure in (A) is aligned and scaled correspondingly to allow for spatial orientation along the z axis. The whole channel pore is wetted throughout the simulation (dewetted regions would appear as white stretches). Although chloride frequently penetrates into the transmembrane pore, sodium does not, again demonstrating the chloride selectivity for this channel.

cryo-EM structure PDB: 3JAE (Du et al., 2015). We applied this protocol to our model of the human α1 GlyR (see the STAR Methods). These restraints were maintained for 150 ns and the final frame was then used to seed three independent production simulations of 300 ns duration. All simulations gave stable open pore configurations, did not exhibit hydrophobic collapse, and exhibited similar pore profiles.

Comparison of the pore radius profiles of our MD structures with the wide-open cryo-EM structure (PDB: 3JAE) shows the mean radius of the MD structures is slightly narrower at the P-2′ ring, but would still allow for the permeation of hydrated chloride ions (Figures 1A and S1; Video S1). The MD structures are thus clearly not in a desensitized state with the P-2′ constriction being 2 Å wider than the currently highest-resolution crystal structure of a desensitized state (human α3 GlyR, PDB: 5TIN; Huang et al., 2017b). Examination of the pore region of a representative simulation structure (the structure with minimal Cα root-mean-square deviation to the average structure)

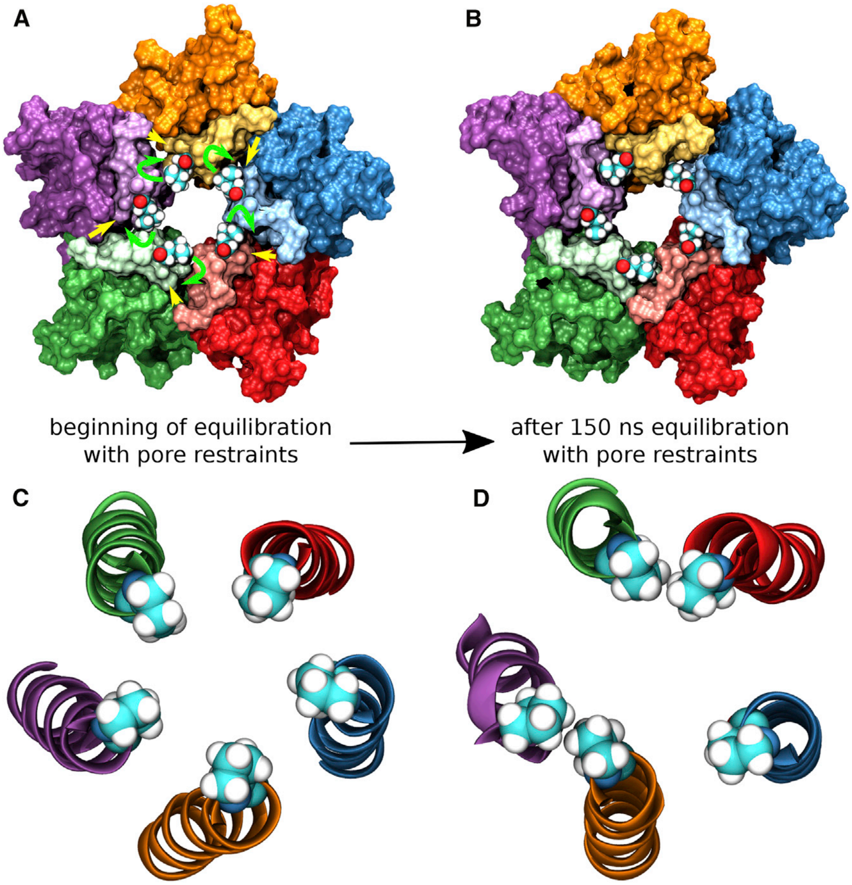

Figure 3. Critical Changes in Pore Geometry

(A and B) View from the extracellular toward the intracellular matrix. The five subunits are colored differently with residues that normally form the hydrophobic binding pocket around 9′ Leu in lighter colors. While at the beginning (A) the five hydrophobic voids at the subunit interfaces are empty and the L9′ residues are orientated slightly more toward the pore center (B), after 150 ns of equilibration with pore restraints the 9′ residues have buried themselves deeply into these hydrophobic pockets and make close contact with the surrounding residues in these pockets, thus firmly locking the structure into an open state conformation.

(C and D) View of the M2 helices with P-2′ gate shown explicitly from the intracellular toward the extracellular side. While at the beginning (C), the structure is 5-fold symmetrical, after 150 ns of equilibration with pore restraints an asymmetric arrangement has been attained (D).

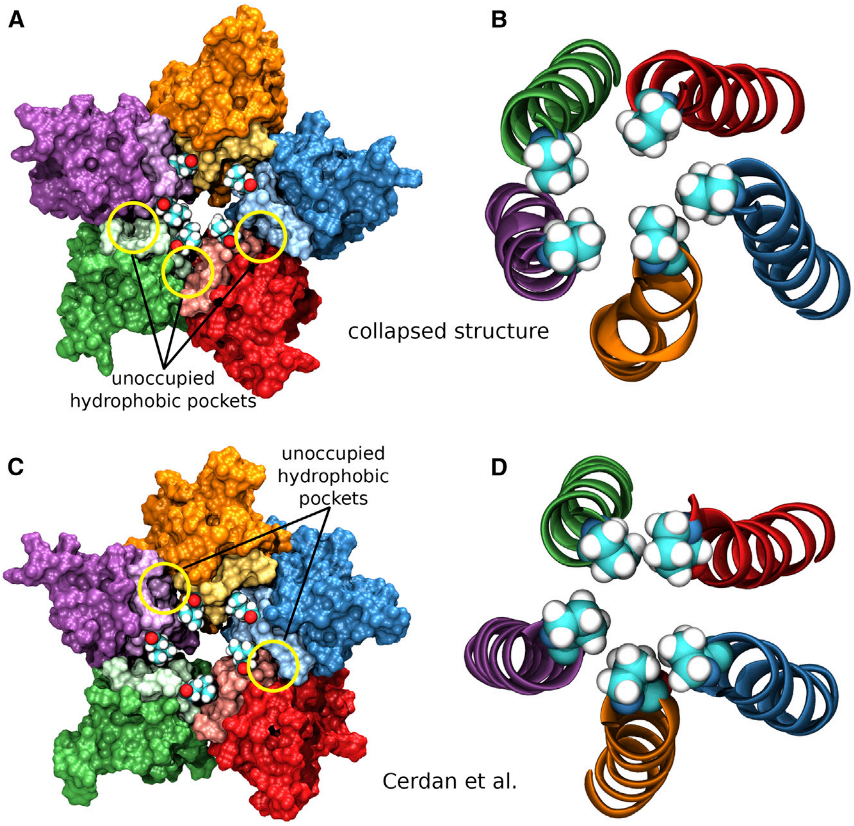

Figure 4. Structural Features of the Collapsed State

(A and B) Representative structure from a collapsed simulation. (A) Although two hydrophobic pockets end up accommodating the corresponding leucine residues, the other three hydrophobic voids undergo a hydrophobic collapse, where the two subunits come closer together and push the leucine residues toward the pore center and decrease the pore radius significantly. (B) With the pore lining M2 helices coming closer together, the P-2′ residues undergo a hydrophobic collapse due to their strong hydrophobic interactions.

(C and D) Representative structure from a simulation by Cerdan et al. (2018). (C) Two hydrophobic pockets fail to accommodate their corresponding L9′ residue, leading to a much smaller pore radius. (D) Without all L9′ gate residues locking the structure into a stable open state, the P-2′ residues come so close to each other that their strong hydrophobic interactions lead to a hydrophobic collapse in the P-2′ ring region as well.

colored by the hydrophobicity of the amino acids surrounding the pore (Figure 1B) revealed that the hydrophobicity was highest in the L9′ region, which is considered the site of a hydrophobic gate in the resting state of the receptor (Filatov and White, 1995; Labarca et al., 1995). Because previous work has suggested that a radius of less than ~4–5 Å in hydrophobic regions can lead to local dehydration and thus be a prohibitive energetic obstacle for ion permeation (Beckstein and Sansom, 2004), we assessed the hydration and ion permeation events.

The ion densities obtained over 300 ns of unrestrained simulation (Figure 2A) reveal that chloride ions can indeed penetrate the transmembrane pore and show selectivity over sodium ions, which preferentially occupy regions in the upper part of the extracellular domain. To resolve the underlying dynamics over time, we analyzed the z coordinates of water, chloride, and sodium ions inside the pore within 5 Å of the ion channel axis (Figure 2B). Chloride ions frequently penetrate the transmembrane pore region, while sodium only rarely accesses this region. Over the 300 ns we count 52 permeation events for chloride and a single permeation event for sodium. In two further repeats, 53 and 49 chloride along with 1 and 0 sodium permeations are observed over 300 ns, respectively (see Figure S2). This is in line with the experimental estimation of a ratio of 28:1 selectivity of chloride over sodium (Keramidas et al., 2002). Furthermore, the whole channel pore is hydrated throughout the simulation (as well as in the other two repeats), demonstrating that the observed pore radius at the hydrophobic L9′ gate is above the threshold for hydrophobic gating.

The Open Conformation Is Stabilized by the Leucine Gate Residues Filling a Hydrophobic Void at the Subunit Interface

Having annotated the receptor state as functionally open, the question that arises is: Why does the structure maintain a conformationally stable open state? The key conformational changes that occur during the equilibration phase with pore restraints are depicted in Figure 3. Taking a cross-section at the level of the L9′ gate reveals that at the beginning of the equilibration phase, there are substantial voids between the subunit interfaces near the leucine residues (Figure 3A). During the equilibration phase, the leucine residues that originally point slightly more toward the pore center, rotate further away from the central axis and bury themselves deeply into these hydrophobic voids and thus lock the subunit interfaces, as well as the neighboring M1 helix, strongly together (Figure 3B; Video S2). With L9′ = L261 being located on the M2 helix of the principal (+) subunit, the corresponding hydrophobic void is formed by I257, V260, and T264 belonging to the M2 helix of the principal (+) subunit, L255, T258, T259, and T262 belonging to the M2 helix of the complementary (−) subunit as well as L233, I234, and L237 of the M1 helix of the complementary (−) subunit (Figure S3). Across the pLGIC superfamily, all the corresponding positions are occupied by hydrophobic residues (Figure S4) and thus the hydrophobic pocket is functionally preserved. This suggests that the open state of the whole superfamily is characterized by the 9′ hydrophobic residues (L or I/V in some cases) being buried in these hydrophobic voids.

We also note that the interaction between the L9′ residue and the methyl group of the T6′ residue of the complementary subunit leads to the T6′ hydroxyl group facing toward the center pore (Figure S3). This makes the profile more hydrophilic at the 6′ ring and thus facilitates pore hydration and ion permeation. Thus, we postulate that the (+) L9′-(−) T6′ hydrophobic

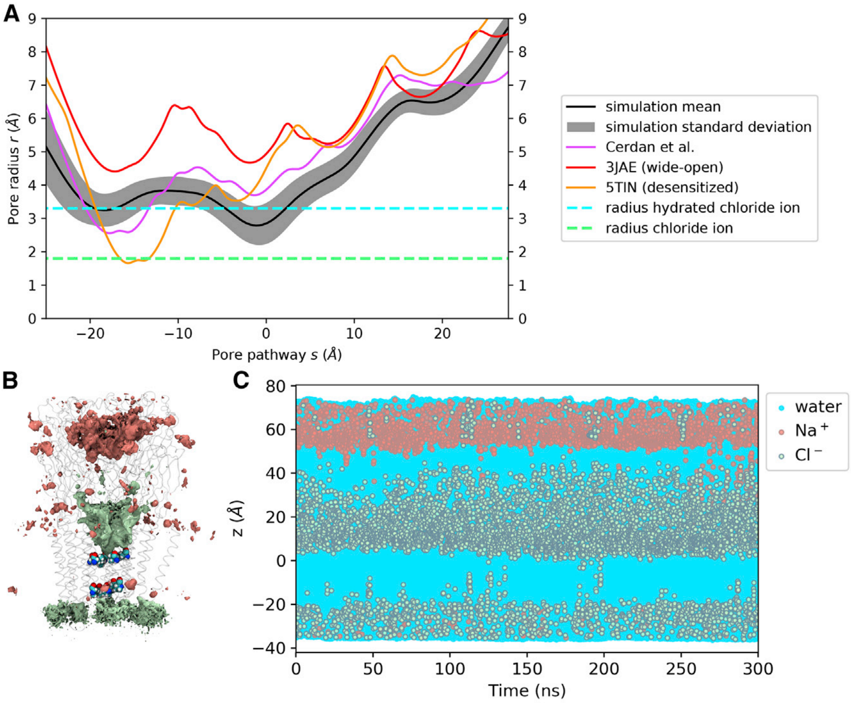

Figure 5. Pore Profile and Ion Permeation of a Hydrophobic Collapsed State

(A) Pore radius profile of a hydrophobic collapse simulation (mean, black; SD, gray) alongside the profile of an MD structure obtained by Cerdan et al. (2018) (purple) as well as the cryo-EM wide-open structure PDB: 3JAE (red) and the desensitized crystal structure with currently highest-resolution PDB: 5TIN (orange). The radii of a dehydrated and a hydrated chloride ion are indicated by dashed green and cyan lines, respectively.

(B) Densities of chloride (green) and sodium (red) ions over a 300-ns production run. The isosurfaces shown represent a density value of 0.5 particles/nm^3. Transparent ribbons indicate the receptor backbone, The L9′ ring and P-2′ ring residues are shown explicitly in van der Waals representation.

(C) Trajectories of water as well as chloride and sodium ion z coordinates within 5 Å of channel axis inside the pore over 300 ns in 150 mM NaCl with no membrane potential (represented by blue, green, and red circles, respectively). The receptor structure in (A) is aligned and scaled correspondingly to allow for spatial orientation along the z axis. The whole channel pore is wetted throughout the simulation (dewetted regions would appear as white stretches), but chloride ions access the transmembrane pore much less than in the stable open state simulation.

interaction plays an important role in the gating mechanism of GlyR. The T6′ position is highly conserved across anion pLGICs (see Figure S4). For anionic pLGIC members, the (+) L/I9′-(−) T6′ interaction could thus be an additional way to fine-tune pore hydration in the gating process.

Although the L9′ residues lock the pore in an open conformation, the P-2′ gate residues rearrange from a symmetric conformation (Figure 3C), which is enforced by the 5-fold symmetry restraints when constructing the cryo-EM model, to an asymmetrical conformation (Figure 3D). In this arrangement, four vicinal proline residues form two pairs via hydrophobic interactions, increasing the distances toward the non-interacting vicinal proline residues, such that the overall asymmetric conformation withstands a hydrophobic collapse.

The Cryo-EM-Based Structure Collapses due to a Sub-optimal Orientation of the Leucine Gate Residues

Having shown that the key to a stable open state structure is the occupation of all five hydrophobic voids at the subunit interface by the L9′ gate residues, it is easy to explain why the original cryo-EM conformation undergoes a hydrophobic collapse. In the original cryo-EM conformation (see Figure 3A), the L9′ residues are not buried in the hydrophobic voids at the subunit interface, but rather point slightly more toward the pore center. In simulations that start from this conformation, the presence of explicit water together with protein dynamic flexibility at a physiological temperature then renders these hydrophobic voids extremely unstable. Collapse usually occurs when two subunits that form a void come closer together and push the corresponding leucine residue toward the pore center (Video S3). It is also possible that a leucine residue randomly ends up in a hydrophobic void. However, the overall collapse is a chaotic process, such that at least two (but usually more) hydrophobic voids collapse with the corresponding leucine residues being pushed toward the pore center, decreasing the pore radius significantly (Figure 4A). The pore lining M2 helices come closer to each other, such that the P-2′ interactions increase, leading to a further collapse of the P-2′ ring (Figure 4B).

A recent report (Cerdan et al., 2018) presented an ion-permeable state of the GlyR obtained from MD simulations. We argue that this state merely corresponds to a collapsed structure. On a structural level, it shows the key features of a collapsed state (see Figures 4C and 4D). At the L9′ gate, two hydrophobic voids are collapsed and the P-2′ ring is also collapsed. The pore radius profile resembles that of our collapsed simulation with the L9′ gate pore radius being slightly larger, but the P-2′ gate pore radius being slightly smaller. The authors reported a single chloride ion permeation event over a total unrestrained simulation time of 500 ns from two repeats. Chloride permeation, however, is not impossible for a collapsed state. Figure 5C illustrates that occasionally chloride ions can penetrate the transmembrane pore region. For the simulation with a collapsing pore presented here, we count 3 chloride ion permeation events over 300 ns. Nevertheless, this is more than an order of magnitude less than the 52 chloride permeation events over 300 ns observed in our stable open state simulation presented here. The chloride ion density obtained from our collapsed simulation has a value below 0.5 particles/nm^3 between the L9′ and the P-2′ gate. Hence, no green isosurface is visible in this region in Figure 5B. In two further simulation repeats undergoing a hydrophobic collapse (see Figures S5 and S6), we observe 3 and 0 chloride permeations over 300 ns unrestrained simulation time, respectively. Considering these results, we conclude that the structure reported by Cerdan et al. does not correspond to a stable open state, but rather to a collapsed state.

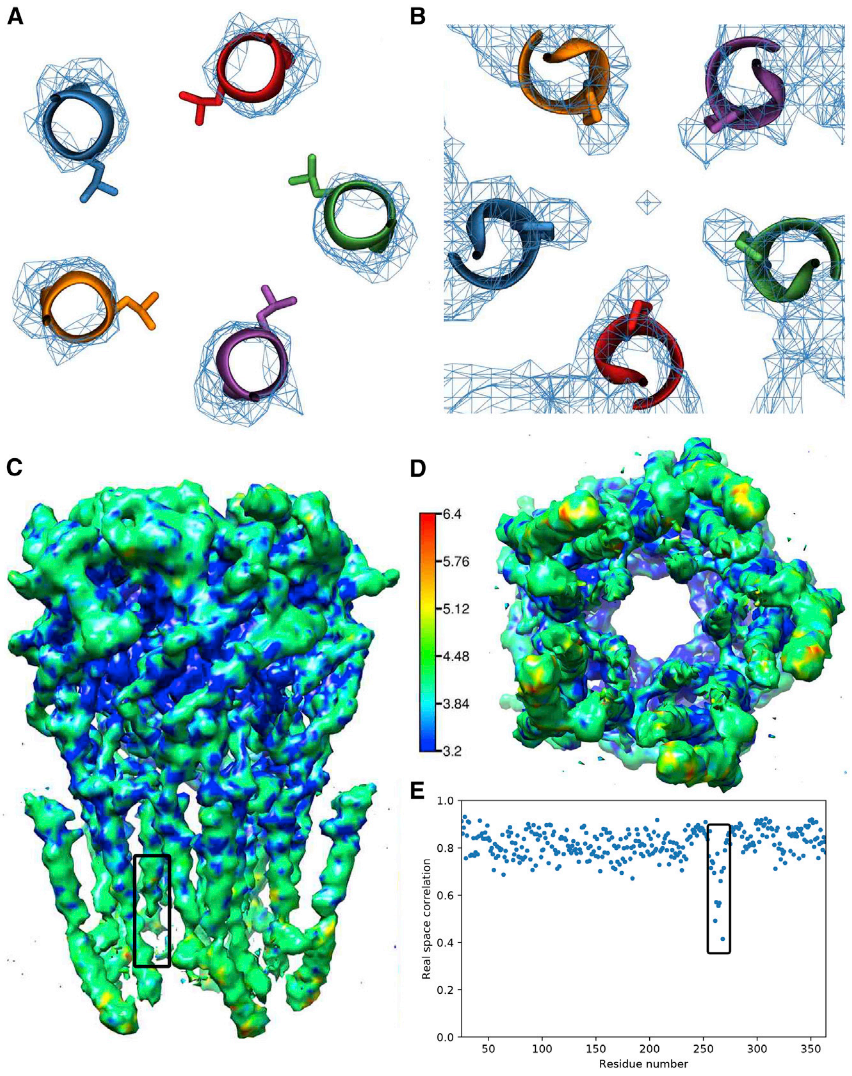

Figure 6. Cryo-EM Density Map and Fitted Model of PDB: 3JAE Structure

(A) Density of the L9′ gate region, contoured at an isosurface with a value of 6.0 together with the fitted model structure for PDB: 3JAE (viewed from the extracellular toward the intracellular side). The density is insufficient to indicate the leucine side-chain orientation, which is therefore purely a result of computational modeling.

(B) Density of the P-2′ gate region, contoured at an isosurface with a value of 5.8 together with the fitted model structure for PDB: 3JAE (viewed from intracellular toward the extracellular side). The density suggests a narrower –2′ pore restriction as the fitted model.

(C and D) Density map colored according to local resolution estimated using RESMAP and visualized with Chimera: side view (C) and view from the intracellular side toward the extracellular side (D). The local resolution is particularly poor in the lower pore lining M2 helix region and its linker to the M1 helix (highlighted by the box) which suggests a higher dynamic flexibility in this region.

(E) Real space correlation between the fitted atomic model and the density map calculated with PHENIX. The correlation is particularly poor in the lower half of the pore lining M2 helix and its linker to the M1 helix (highlighted by black box), indicating that the quality of the atomic model is particularly poor in this region.

DISCUSSION

Simulations of open states of the pLGIC superfamily undergo a hydrophobic collapse once artificial restraints on the protein structure are released (Cheng and Coalson, 2012; Cerdan et al., 2018; Calimet et al., 2013; Yoluk et al., 2015). This is why it has become common practice to apply restraints on the protein backbone to artificially fix its position (Basak et al., 2018; Trick et al., 2016; Gonzalez-Gutierrez et al., 2017). Cross-pore restraints have been used before to restrain the subsection of the channel near the hydrophobic gate (LeBard et al., 2012; Chiodo et al., 2015). Interestingly, Chiodo et al. (2015) observed a stable hydrated state of a homology model of the nicotinic acetylcholine receptor, upon the release of such restraints. However, although they observed water permeation through the channel, they were unable to observe a single ion permeation event in the course of 800 ns (aggregated) simulation time. A similar lack of ion permeation was also reported for an open state model of the GluCl channel (Cheng and Coalson, 2012). Thus, it is unclear to what extent those studies capture a genuine open state. The GLIC channel is a prokaryotic member of the Cys-loop superfamily and a structure exists which has been annotated as an apparently open state. A comparison of our open state with the PDB: 3EAM structure (Bocquet et al., 2009) shows that the isoleucine of GLIC at the equivalent 9′ position, is pointing slightly more toward the channel pore. However, this structure is stabilized in a non-physiological way by non-polar regions of detergents molecules, which are trapped inside the pore and exclude water in this region. Thus, a direct meaningful comparison here is difficult.

Recently, Polovinkin et al. (2018) reported that while simulations starting from one structure of the 5-HT_3 receptor (I2, PDB: 6HIQ) gave the familiar hydrophobic collapse, simulations starting from another (F, PDB: 6HIN) allowed ions and water to flow throughout the trajectory and that wetting was correlated to rotation of L9′ out of the pore lumen. The PDB: 6HIN structure shows that the L9′ leucines are indeed rotated away from the pore axis (Figure S7), consistent with our refined model of the GlyR and supporting the view that this corresponds to an open state. By forming strong hydrophobic interactions with residues of the M2 and M1 transmembrane helices of the complementary subunit, the leucines at L9′ lock the pore into a stable open conformation.

It is insightful in this context to revisit the original density map of the wide-open PDB: 3JAE structure from cryo-EM (Du et al., 2015) paying particular attention to the areas of the key conformational changes that stabilize the open state, namely the L9′ and P-2′ region. Close inspection shows no density for the orientation of the L9′ gate side chains (Figure 6A), meaning that their

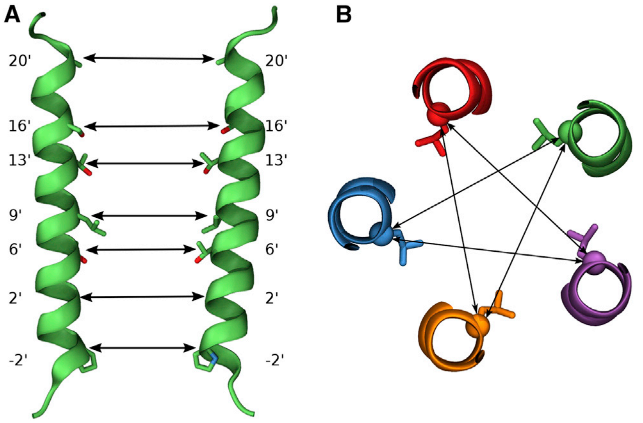

Figure 7. Pore Restraints

(A) Two non-adjacent pore lining M2 helices with seven pore lining residues. The double arrows indicate the pore cross distances on which the flat bottom pore restraints act.

(B) Illustration of the pore cross distances at the L9′ gate (view from the extracellular toward the intracellular side). In total, there are five cross distances for the 9′ ring. The cross distances for the other pore lining residues are not shown for clarity, but, similarly, there are five cross distances per pore lining residue position, so that in total 7 × 5 = 35 cross distances are restrained.

Table 1. Initial Equilibration Protocol

Time	Ensemble	Position Restraints On	Force Constant (kJ mol^{-1} nm$^{-1)}$
100 ps	NVT	protein heavy atoms, ligand heavy atoms, crystal water oxygen	1,000
1 ns	NPT	protein heavy atoms, ligand heavy atoms, crystal water oxygen	1,000
10 ns	NPT	protein backbone, ligand Cα atom, crystal water oxygen	1,024
1 ns	NPT	protein backbone, ligand Cα atom, crystal water oxygen	512
1 ns	NPT	protein backbone, ligand Cα atom, crystal water oxygen	256
1 ns	NPT	protein backbone, ligand Cα atom, crystal water oxygen	128
1 ns	NPT	protein backbone, ligand Cα atom, crystal water oxygen	64

atomic coordinates are purely a result of computational modeling and that alternative leucine side-chain conformations are equally probable.

Inspection of the density at the P-2′ gate region also shows that the map is of particularly poor resolution in this region (Figures 6C and 6D) and likely corresponds to the high mobility of this region (Du et al., 2015). The data would be consistent with an ensemble of asymmetric P-2′ gate conformations, which, when averaged, result in a symmetric distribution of density. Consistent with this, our simulations confirm a higher mobility in this area, as measured by the root-mean-square fluctuation (RMSF) of Cα atoms (Figure S8). While typical RMSF values for Cα atoms in α helices are around 0.7 Å, these values are significantly increased near the P-2′ gate (>1 Å). This means more conformational diversity of the intracellular pore mouth is accessible at a physiological temperature of 37°C. What we observe in the simulations, is that during two of the simulation runs, the prolines are highly dynamic yet remain as two pairs and one lone proline. In the third simulation, a lone proline starts to interact with a pair and the simulation proceeds as an arrangement of three and two prolines, thus maintaining a dynamic asymmetry. The density map in the P-2′ ring region shows that there is density for proline further toward the pore center (Figure 6B), suggesting that on average the pore radius might in reality be ~1 Å or so smaller than the unusual wide-open -2′ Pro radius of the modeled atomic structure. This is an interesting discrepancy between the density map and the fitted model, because our simulations also suggest a smaller P-2′ pore radius of comparable magnitude (see Figure 1A). Moreover, the real space correlation between the modeled structure for PDB: 3JAE and the density map is particularly meagre in this region, with R0′ peaking with a very low correlation below 0.4 (Figure 6E). This means that the atomic model PDB: 3JAE is of poor quality in the M1-M2 linker and the lower M2 region, where the P-2′ gate is located.

In conclusion, the results presented here demonstrate the potential of MD simulations to refine structures of channel proteins derived from cryo-EM or X-ray crystallography and highlight the potential issues surrounding them. MD has the power to help resolve these problems and indeed flexible fitting methods (Igaev et al., 2019), seem like a promising way forward to use this more directly at the model-building stage. In the case of the pLGIC superfamily, the question of what truly represents the physiological open state has been subject to debate since 2015. Although it is clear that the observation of collapsed, mostly non-conducting, states are not likely representative of the true open state, it does raise the question of whether these collapsed states are in any way physiologically relevant. It may still be the case that they represent some kind of intermediate that is part of the gating process (including, for example, recovering from desensitization). If this was the case, the gating process would not be a concerted mechanism. To resolve that is likely to require substantial further work.

In this work, we report an MD-refined open and ion-conductive structure of the GlyR as a representative open state of the Cys-loop family. In light of our simulation results, and in revisiting the original cryo-EM density map together with the fitted atomic model PDB: 3JAE, we provide a different interpretation of the experimental data. We argue that the open state is stabilized by the L9′ residues, whose orientation is not resolved in the cryo-EM density map, being buried into hydrophobic pockets at the subunit interfaces, thus locking the receptor in an open state at the 9′ ring. We also suggest that the poor resolution of the cryo-EM map at the P-2′ ring is the result of high dynamic flexibility in this region, and the open state corresponds to an (on average symmetric) ensemble of possible asymmetric arrangements of the proline residues. Moreover, we argue that the -2′ constriction is ~1 Å smaller in radius than the fitted atomic

model PDB: 3JAE suggests, which is underpinned by the original cryo-EM density in this region as well as our MD simulation results and would be more in line with other open structures of the pLGIC superfamily. We note that the proline at the -2′ position is not conserved among other members of the Cys-loop family, and whether the equivalent residues behave in a similar manner remains to be seen. On the other hand, the non-polar 9′ position is highly conserved across the pLGICs (L or occasionally I/V), as are the hydrophobic pockets. Thus, the hydrophobic lock mechanism reported here is likely to play a key role in gating for the whole superfamily.

STAR★METHODS

Detailed methods are provided in the online version of this paper and include the following:

- KEY RESOURCES TABLE
- LEAD CONTACT AND MATERIALS AVAILABILITY
- METHOD DETAILS
  - Homology Modelling
  - Ligand Positioning and Parameterization
  - System Set Up
  - All Atom Molecular Dynamics (MD) Simulations
  - Analysis and Visualization
- QUANTIFICATION AND STATISTICAL ANALYSIS
- DATA AND CODE AVAILABILITY

SUPPLEMENTAL INFORMATION

Supplemental Information can be found online at https://doi.org/10.1016/j.str.2019.10.019.

ACKNOWLEDGMENTS

We thank our colleagues for useful discussions and analysis scripts, especially Hannah Smith, Gianni Klesse and Shanlin Rao. MAD received funding by the Oxford Wolfson Marriott Graduate Scholarship in Biochemistry, the Studienstiftung des Deutschen Volkes, the Goodger and Schorstein Research Scholarship in Medical Sciences as well as the Vice-Chancellors' fund. This project made use of computation time on JADE (EP/P020275/1) via HECBioSim (http://www.hecbiosim.ac.uk), supported by EPSRC (grant no. EP/R029407/1). PCB thanks the MRC for funding (BB/S001247/1).

AUTHOR CONTRIBUTIONS

P.C.B. and M.A.D. conceived the study. M.A.D. designed the simulation protocol, performed and analyzed the MD simulations, as well as the analysis of the cryo-EM map and atomic model PDB: 3JAE. M.A.D. and P.C.B. interpreted the results and wrote the paper.

DECLARATION OF INTERESTS

The authors have no financial interests to declare, no positions to declare. The authors have no related patents to declare.

Received: July 4, 2019
Revised: October 11, 2019
Accepted: October 30, 2019
Published: November 18, 2019

REFERENCES

Abraham, M.J., Murtola, T., Schulz, R., Páll, S., Smith, J.C., Hess, B., and Lindahl, E. (2015). GROMACS: high performance molecular simulations through multi-level parallelism from laptops to supercomputers. SoftwareX *1–2*, 19–25.

Adams, P.D., Afonine, P.V., Bunkoczi, G., Chen, V.B., Davis, I.W., Echols, N., Headd, J.J., Hung, L.-W., Kapral, G.J., Grosse-Kunstleve, R.W., et al. (2010). PHENIX: a comprehensive Python-based system for macromolecular structure solution. Acta Crystallogr. D Biol. Crystallogr. *66*, 213–221.

Aryal, P., Sansom, M.S.P., and Tucker, S.J. (2015). Hydrophobic gating in ion channels. J. Mol. Biol. *427*, 121–130.

Basak, S., Gicheru, Y., Rao, S., Sansom, M.S.P., and Chakrapani, S. (2018). Cryo-EM reveals two distinct serotonin-bound conformations of full-length 5-HT3A receptor. Nature *563*, 270–274.

Beckstein, O., Biggin, P.C., and Sansom, M.S.P. (2001). A hydrophobic gating mechanism for nanopores. J. Phys. Chem. B *105*, 12902–12905.

Beckstein, O., and Sansom, M.S.P. (2004). The influence of geometry, surface character, and flexibility on the permeation of ions and water through biological pores. Phys. Biol. *1*, 42–52.

Beckstein, O., and Sansom, M.S.P. (2006). A hydrophobic gate in an ion channel: the closed state of the nicotinic acetylcholine receptor. Phys. Biol. *3*, 147–159.

Benkert, P., Kunzli, M., and Schwede, T. (2009). QMEAN server for protein model quality estimation. Nucleic Acids Res. *37*, W510–W514.

Berendsen, H.J.C., Postma, J.P.M., van Gunsteren, W.F., DiNola, A., and Haak, J.R. (1984). Molecular dynamics with coupling to an external bath. J. Chem. Phys. *81*, 3684–3690.

Bocquet, N., Nury, H., Baaden, M., Le Poupon, C., Changeux, J.-P., Delarue, M., and Corringer, P.-J. (2009). X-ray structure of a pentameric ligand-gated ion channel in an apparently open conformation. Nature *457*, 111–114.

Bode, A., and Lynch, J. (2014). The impact of human hyperekplexia mutations on glycine receptor structure and function. Mol. Brain *7*, 2.

Burgos, C.F., Yévenes, G.E., and Aguayo, L.G. (2016). Structure and pharmacologic modulation of inhibitory glycine receptors. Mol. Pharmacol. *90*, 318.

Bussi, G., Donadio, D., and Parrinello, M. (2007). Canonical sampling through velocity rescaling. J. Chem. Phys. *126*, 014101.

Calimet, N., Simoes, M., Changeux, J.-P., Karplus, M., Taly, A., and Cecchini, M. (2013). A gating mechanism of pentameric ligand-gated ion channels. Proc. Natl. Acad. Sci. U S A *110*, E3987–E3996.

Cecchini, M., and Changeux, J.-P. (2015). The nicotinic acetylcholine receptor and its prokaryotic homologues: structure, conformational transitions and allosteric modulation. Neuropharmacology *96* (Part B), 137–149.

Cerdan, A.H., Martin, N.É., and Cecchini, M. (2018). An ion-permeable state of the glycine receptor captured by molecular dynamics. Structure *26*, 1555–1562.

Cheng, M.H., and Coalson, R.D. (2012). Energetics and ion permeation characteristics in a glutamate-gated chloride (GluCl) receptor channel. J. Phys. Chem. B *116*, 13637–13643.

Chiodo, L., Malliavin, T.E., Maragliano, L., Cottone, G., and Ciccotti, G. (2015). A structural model of the human α7 nicotinic receptor in an open conformation. PLoS One *10*, e0133011.

Darden, T., York, D., and Pedersen, L. (1993). Particle mesh Ewald—an N.log(N) method for Ewald sums in large systems. J. Chem. Phys. *98*, 10089–10092.

de Jong, D.H., Baoukina, S., Ingólfsson, H.I., and Marrink, S.J. (2016). Martini straight: boosting performance using a shorter cutoff and GPUs. Comput. Phys. Commun. *199*, 1–7.

de Jong, D.H., Singh, G., Bennett, W.F.D., Arnarez, C., Wassenaar, T.A., Schäfer, L.V., Periole, X., Tieleman, D.P., and Marrink, S.J. (2013). Improved parameters for the Martini coarse-grained protein force field. J. Chem. Theory Comput. *9*, 687–697.

Du, J., Lu, W., Wu, S., Cheng, Y., and Gouaux, E. (2015). Glycine receptor mechanism elucidated by electron cryo-microscopy. Nature *526*, 224–229.

Edgar, R.C. (2004). MUSCLE: multiple sequence alignment with high accuracy and high throughput. Nucleic Acids Res. *32*, 1792–1797.

Essman, U., Perera, L., Berkowitz, M.L., Darden, T., Lee, H., and Pedersen, L.G. (1995). A smooth particle mesh Ewald method. J. Chem. Phys. *103*, 8577–8593.

Filatov, G.N., and White, M.M. (1995). The role of conserved leucines in the M2 domain of the acetylcholine-receptor in channel gating. Mol. Pharmacol. *48*, 379–384.

Gonzalez-Gutierrez, G., Wang, Y., Cymes, G.D., Tajkhorshid, E., and Grosman, C. (2017). Chasing the open-state structure of pentameric ligand-gated ion channels. J. Gen. Physiol. *149*, 1119.

Gotti, C., Zoli, M., and Clementi, F. (2006). Brain nicotinic acetylcholine receptors: native subtypes and their relevance. Trends Pharmacol. Sci. *27*, 482–491.

Hess, B., Bekker, J., Berendsen, H.J.C., and Fraaije, J.G.E.M. (1997). LINCS: a linear constraint solver for molecular simulations. J. Comput. Chem. *18*, 1463–1472.

Hibbs, R., and Gouaux, E. (2011). Principles of activation and permeation in an anion-selective Cys-loop receptor. Nature *474*, 54–60.

Horn, A.H.C. (2014). A consistent force field parameter set for zwitterionic amino acid residues. J. Mol. Model. *20*, 2478.

Huang, X., Chen, H., Michelsen, K., Schneider, S., and Shaffer, P.L. (2015). Crystal structure of human glycine receptor alpha3 bound to antagonist strychnine. Nature *526*, 277–280.

Huang, X., Chen, H., and Shaffer, P.L. (2017a). Crystal structures of human GlyRα3 bound to ivermectin. Structure *25*, 945–950.e2.

Huang, X., Shaffer, P.L., Ayube, S., Bregman, H., Chen, H., Lehto, S.G., Luther, J.A., Matson, D.J., McDonough, S.I., Michelsen, K., et al. (2017b). Crystal structures of human glycine receptor α3 bound to a novel class of analgesic potentiators. Nat. Struct. Mol. Biol. *24*, 108–113.

Humphrey, W., Dalke, A., and Schulten, K. (1996). VMD—visual molecular dynamics. J. Mol. Graph. *14*, 33–38.

Igaev, M., Kutzner, C., Bock, L.V., Vaiana, A.C., and Grubmüller, H. (2019). Automated cryo-EM structure refinement using correlation-driven molecular dynamics. Elife *8*, e43542.

Jämbeck, J.P.M., and Lyubartsev, A.P. (2012a). Derivation and systematic validation of a refined all-atom force field for phosphatidylcholine lipids. J. Phys. Chem. B *116*, 3164–3179.

Jämbeck, J.P.M., and Lyubartsev, A.P. (2012b). An extension and further validation of an all-atomistic force field for biological membranes. J. Chem. Theory Comput. *8*, 2938–2948.

Jämbeck, J.P.M., and Lyubartsev, A.P. (2013). Another piece of the membrane puzzle: extending Slipids further. J. Chem. Theory Comput. *9*, 774–784.

Jensen, A.A., Frølund, B., Liljefors, T., and Krogsgaard-Larsen, P. (2005). Neuronal nicotinic acetylcholine receptors: structural revelations, target identifications and therapeutic inspirations. J. Med. Chem. *48*, 4705–4745.

Jorgensen, W.L., Chandresekhar, J., Madura, J.D., Impey, R.W., and Klein, M.L. (1983). Comparison of simple potential functions for simulating liquid water. J. Chem. Phys. *79*, 926–935.

Katz, B., and Thesleff, S. (1957). A study of the densitization produced by acetylcholine at the motor end-plate. J. Physiol. *138*, 63–80.

Keramidas, A., Moorhouse, A.J., Pierce, K.D., Schofield, P.R., and Barry, P.H. (2002). Cation-selective mutations in the M2 domain of the inhibitory glycine receptor channel reveal determinants of ion-charge selectivity. J. Gen. Physiol. *119*, 393.

Klesse, G., Rao, S., Sansom, M.S.P., and Tucker, S.J. (2019). CHAP: a versatile tool for the structural and functional annotation of ion channel pores. J. Mol. Biol. *431*, 3353–3365.

Kucukelbir, A., Sigworth, F.J., and Tagare, H.D. (2014). Quantifying the local resolution of cryo-EM density maps. Nat. Methods *11*, 63–65.

Labarca, C., Nowak, M.W., Zhang, H., Tang, L., Desphande, P., and Lester, H.A. (1995). Channel gating governed symmetrically by conserved leucine residues in the M2 domain of nicotinic receptors. Nature *376*, 514–516.

Lape, R., Colquhoun, D., and Sivilotti, L.G. (2008). On the nature of partial agonism in the nicotinic receptor superfamily. Nature *454*, 722–727.

Lape, R., Plested, A.J.R., Moroni, M., Colquhoun, D., and Sivilotti, L.G. (2012). The α1K276E startle disease mutation reveals multiple intermediate states in the gating of glycine receptors. J. Neurosci. *32*, 1336–1352.

LeBard, D.N., Hénin, J., Eckenhoff, R.G., Klein, M.L., and Brannigan, G. (2012). General anesthetics predicted to block the GLIC pore with micromolar affinity. PLoS Comput. Biol. *8*, e1002532.

Lindorff-Larsen, K., Piana, S., Palmo, K., Maragakis, P., Klepeis, J., Dror, R., and Shaw, D. (2010). Improved side-chain torsion potentials for the Amber ff99SB protein force field. Proteins *78*, 1950–1958.

Lynch, J.W. (2004). Molecular structure and function of the glycine receptor chloride channel. Physiol. Rev. *84*, 1051–1095.

Lynch, J.W. (2009). Native glycine receptor subtypes and their physiological roles. Neuropharmacology *56*, 303–309.

Lynch, J.W., and Callister, R.J. (2006). Glycine receptors: a new therapeutic target in pain pathways. Curr. Opin. Investig. Drugs *7*, 48–53.

Lynch, J.W., Zhang, Y., Talwar, S., and Estrada-Mondragon, A. (2017). Glycine receptor drug discovery. Adv. Pharmacol. *79*, 225–253.

Michaud-Agrawal, N., Denning, E.J., Woolf, T., and Beckstein, O. (2011). MDAnalysis: a toolkit for the analysis of molecular dynamics simulations. J. Comput. Chem. *32*, 2319–2327.

Nemecz, Á., Prevost Marie, S., Menny, A., and Corringer, P.-J. (2016). Emerging molecular mechanisms of signal transduction in pentameric ligand-gated ion channels. Neuron *90*, 452–470.

Nys, M., Kesters, D., and Ulens, C. (2013). Structural insights into Cys-loop receptor function and ligand recognition. Biochem. Pharmacol. *86*, 1042–1053.

Parinello, M., and Rahman, A. (1981). Polymorphic transitions in single crystals—a new molecular dynamics method. J. Appl. Phys. *52*, 7182–7190.

Pettersen, E.F., Goddard, T.D., Huang, C.C., Couch, G.S., Greenblatt, D.M., Meng, E.C., and Ferrin, T.E. (2004). USCF chimera—a visualization system for exploratory research and analysis. J. Comput. Chem. *25*, 1605–1612.

Polovinkin, L., Hassaine, G., Perot, J., Neumann, E., Jensen, A.A., Lefebvre, S.N., Corringer, P.-J., Neyton, J., Chipot, C., Dehez, F., et al. (2018). Conformational transitions of the serotonin 5-HT3 receptor. Nature *563*, 275–279.

Safar, F., Hurdiss, E., Erotocritou, M., Greiner, T., Lape, R., Irvine, M.W., Fang, G., Jane, D., Yu, R., Dämgen, M.A., et al. (2017). The startle disease mutation E103K impairs activation of human homomeric α1 glycine receptors by disrupting an intersubunit salt bridge across the agonist binding site. J. Biol. Chem. *292*, 5031–5042.

Sauguet, L., Poitevin, F., Murail, S., Van Renterghem, C., Moraga-Cid, G., Malherbe, L., Thompson, A.W., Koehl, P., Corringer, P.J., Baaden, M., and Delarue, M. (2013). Structural basis for ion permeation mechanism in pentameric ligand-gated ion channels. EMBO J. *32*, 728.

Shen, M.Y., and Sali, A. (2006). Statistical potential for assessment and prediction of protein structures. Protein Sci. *15*, 2507–2524.

Thompson, A.J., Lester, H.A., and Lummis, S.C.R. (2010). The structural basis of function in Cys-loop receptors. Q. Rev. Biophys. *43*, 449–499.

Trick, J.L., Chelvaniththilan, S., Klesse, G., Aryal, P., Wallace, E.J., Tucker, S.J., and Sansom, M.S.P. (2016). Functional annotation of ion channel structures by molecular simulation. Structure *24*, 2207–2216.

Van Arnam, E.B., and Dougherty, D.A. (2014). Functional probes of drug-receptor interactions implicated by structural studies: Cys-loop receptors provide a fertile testing ground. J. Med. Chem. *57*, 6289–6300.

Wassenaar, T.A., Ingólfsson, H.I., Böckmann, R.A., Tieleman, D.P., and Marrink, S.J. (2015). Computational lipidomics with insane: a versatile tool for generating custom membranes for molecular simulations. J. Chem. Theory Comput. *11*, 2144–2155.

Wassenaar, T.A., Pluhackova, K., Böckmann, R.A., Marrink, S.J., and Tieleman, D.P. (2014). Going backward: a flexible geometric approach to reverse transformation from coarse grained to atomistic models. J. Chem. Theory Comput. *10*, 676–690.

Webb, B., and Sali, A. (2014). Comparative protein structure modeling using modeller. Curr. Protoc. Bioinformatics *5*, 5.6.1–5.6.32.

Wimley, C.W., and White, S.H. (1996). Experimentally determined hydrophobicity scale for proteins at membrane interfaces. Nat. Struct. Biol. *3*, 842–848.

Wimley, W.C., Creamer, T.P., and White, S.H. (1996). Solvation energies of amino acid side chains and backbone in a family of host-guest pentapeptides. Biochemistry *35*, 5109–5124.

Yoluk, O., Lindahl, E., and Andersson, M. (2015). Conformational gating dynamics in the GluCl anion-selective chloride channel. ACS Chem. Neurosci. *6*, 1459–1467.

Yu, R., Hurdiss, E., Greiner, T., Lape, R., Sivilotti, L., and Biggin, P.C. (2014). Agonist and antagonist binding in human glycine receptors. Biochemistry *53*, 6041–6051.

STAR★METHODS

KEY RESOURCES TABLE

REAGENT or RESOURCE	SOURCE	IDENTIFIER
Deposited Data		
First frame of MD trajectory (.gro)	This work	10.5281/zenodo.3476169
Representative open state (.pdb)	This work	10.5281/zenodo.3476169
Trajectory of stable open state	This work	10.5281/zenodo.3476169
GlyR α1 +Gly	(Du et al., 2015)	PDB: 3JAE
X-ray structure of GlyR α3 +Gly	Huang et al., 2017b	PDB:5TIN
Cryo-EM structure of 5-HT3 receptor	Polovinkin et al., 2018	PDB:6HIN
Open structure of GLIC	Bocquet et al., 2009	PDB:3EAM
Software and Algorithms		
AMBER99SB-ILDN force-field	Lindorff-Larsen et al., 2010	http://ambermd.org/AmberModels.php
Slipids force-field	Jämbeck and Lyubartsev, 2012a, 2012b	http://www.fos.su.se/~sasha/SLipids/
Zwitterionic glycine force-field	Horn, 2014	http://research.bmh.manchester.ac.uk/bryce/amber
CHAP	Klesse et al., 2019	https://www.channotation.org
gromacs	Abraham et al., 2015	http://www.gromacs.org/
MDAnalysis	Michaud-Agrawal et al., 2011	https://www.mdanalysis.org/
MODELLER 9.16	Webb and Sali, 2014	https://salilab.org/modeller/
Phenix	Adams et al., 2010	https://www.phenix-online.org/
RESMAP	Kucukelbir et al., 2014	http://resmap.sourceforge.net
UCSF Chimera	Pettersen et al., 2004	https://www.cgl.ucsf.edu/chimera/
VMD 1.9	Humphrey et al., 1996	http://www.ks.uiuc.edu/Research/vmd/

LEAD CONTACT AND MATERIALS AVAILABILITY

Further information and requests for resources and reagents should be directed to and will be fulfilled by the Lead Contact, Philip Biggin (Philip.biggin@bioch.ox.ac.uk).

All production run trajectories generated in this study are available from the Lead Contact without restriction.

METHOD DETAILS

Homology Modelling

The human α1 GlyR model was constructed from the structure of *Danio rerio* α1 GlyR (glycine-bound open state; PDB: 3JAE (Du et al., 2015)) as previously reported (Safar et al., 2017). Briefly sequences were aligned using Muscle (Edgar, 2004) and adjusted manually. MODELLER version 9.16 (Webb and Sali, 2014) was used to generate 100 models and from of the intersection of the top 10 of both molecular PDF and DOPE score (Shen and Sali, 2006), the model with the best QMEAN score (Benkert et al., 2009) was chosen for simulation. Hydrogens were added to the protein with the pdb2gmx tool of Gromacs 5.1 (Abraham et al., 2015), with the default protonation state at pH = 7.4.

Ligand Positioning and Parameterization

Glycine was positioned into all 5 orthosteric binding sites according to its position in the currently highest resolution glycine receptor structure; PDB:5TIN (Huang et al., 2017b), 2.6 Å resolution (note that glycine was bound to the receptor but no density was discernible in PDB:3JAE (Du et al., 2015), 3.9 Å resolution). We used the parameterization by Horn (Horn, 2014) for zwitterionic amino acids with the AMBER99SB-ILDN force field. We also included a water molecule in the binding pocket which is resolved in PDB:5TIN and shown to be stable in MD-simulations (Safar et al., 2017; Yu et al., 2014).

System Set Up

Systems were set up using a coarse-grained approach with the Martini force field version 2.2 using martinize (de Jong et al., 2013) to coarse-grain the protein model and the "insane" programme (Wassenaar et al., 2015), using a hexagonal prism as the periodic simulation box. The protein was embedded in a 1-palmitoyl-2-oleoyl-sn-glycero-phosphocholine (POPC) bilayer, solvated in water and

sodium and chloride ions were added to neutralise the net charge and simulate a physiological concentration of 150 mM. Gromacs 2018 (Abraham et al., 2015) was used as molecular dynamics engine for all simulations. After energy minimisation, the system was equilibrated at 310 K and 1 bar for 10 ns. Simulation details were based on the recommended new-rf.mdp parameters (de Jong et al., 2016) and appropriately adjusted. The final frame was then converted to all-atom resolution via the programme "backward" (Wassenaar et al., 2014). The back-mapped receptor was replaced by the original all-atom model with the glycine ligands and stable water molecules bound, resulting in a total of 136,334 atoms.

All Atom Molecular Dynamics (MD) Simulations

Each system was energy-minimised using the steepest descent algorithm until the maximum force fell below a tolerance of 1000 kJ mol^{-1} nm^{-1}. The initial equilibration protocol is summarised in Table 1. The final frame after this initial equilibration period was used as the starting point for three independent simulations with different velocities, all of which exhibited hydrophobic collapse. To find a stable open channel structure, we extended the initial equilibration as follows. The ion channel pore was kept open via flat-bottom harmonic cross distance position restraints between the Cα atoms of pore-lining residues (pore restraints, see Figure 7). The flat bottom potential only acts when the Cα atoms of non-adjacent pore-lining residues come closer to each other than their distance in the open-state cryo-EM structure 3JAE with a force proportional to the difference in distance (5000 kJ mol^{-1} nm^{-1}). This keeps the channel pore from collapsing initially and gives it enough freedom to find a stable configuration. This additional restrained simulation step with pore restraints was performed for 150 ns. The final frame was used as the starting configuration for three independent repeats, initiated with different velocities. All three repeats showed a stable open channel configuration. During both equilibration and production runs, flat-bottomed restraint terms were applied to the free glycine ligands in the binding site to prevent their dissociation, but still allow reasonable exploration of the pocket. These restraints act if the distance between Cα atoms of key residues in the binding site and the Cα atom of the glycine ligand exceed a threshold distance (namely, 10.60 Å to the (+)F207 Cα, 10.53 Å to the (-)F63 Cα and 9.15 Å to the (-)S129 Cα, with a force constant of 5000 kJ mol^{-1} nm^{-1}).

The force fields used were AMBER99SB-ILDN (Lindorff-Larsen et al., 2010) for protein and ions, TIP3P for water (Jorgensen et al., 1983), Slipids (Jämbeck and Lyubartsev, 2012a, 2012b, 2013) for lipids and the parameters for zwitterionic amino acids by Horn (Horn, 2014) for the glycine ligands. Periodic boundary conditions in all three spatial dimensions were applied to mimic bulk conditions. The Bussi thermostat (Bussi et al., 2007) was used to maintain the temperature constant at physiological 310 K using a coupling constant of 0.1ps. In all simulations with constant pressure, a barostat was coupled to the system in a semi-isotropic manner to maintain a pressure of 1 bar. The time constant used for pressure coupling was 1 ps and the isothermal compressibility set to 4.5 × 10-5 bar^{-1}. For the initial equilibration, the Berendsen barostat (Berendsen et al., 1984) was used and the Parrinello-Rahmann barostat (Parinello and Rahman, 1981) for production runs as well as the simulation period with pore restraints. The van der Waals interactions were cut off at 10 Å and a dispersion correction was applied to energy and pressure. Electrostatic interactions were treated using the smooth Particle Mesh Ewald (PME) method (Essman et al., 1995; Darden et al., 1993), where the real space contribution was cut off at 10 Å and the reciprocal energy term obtained in k-space was calculated on a grid with 1.2 Å spacing using 4th order B-splines for interpolation. The Verlet cut-off scheme was used to generate a pair-list with buffering using a tolerance of 0.005 kJ/mol/ps for pair-interactions per particle. All bonds involving hydrogens were constrained with the LINCS algorithm (Hess et al., 1997), allowing for a time step of 2 fs (except in the first dynamics run (NVT-equilibration), where a shorter time step of 1 fs was used). Coordinates were written out every 10 ps.

Analysis and Visualization

Pore profiles, volumes and hydrophobicity were calculated with CHAP (Klesse et al., 2019). Hydrophobicity was calculated according to the Wimley and White scale (Wimley et al., 1996; Wimley and White, 1996). Trajectory analysis was performed with MDAnalysis (Michaud-Agrawal et al., 2011) and in-house scripts. The local resolution of the cryo-EM density map was estimated with RESMAP (Kucukelbir et al., 2014), the real space correlation between atomic model and map was calculated with Phenix (Adams et al., 2010). Visualization was performed in VMD (Humphrey et al., 1996) and UCSF Chimera (Pettersen et al., 2004).

QUANTIFICATION AND STATISTICAL ANALYSIS

Pore radius profiles are reported as mean and 1 standard deviation over 300 equally distributed data points per 300ns.

DATA AND CODE AVAILABILITY

The following are accessible via zenodo.org via the https://doi.org/10.5281/zenodo.3476169.

File Name: md_fit.gro.
Description: The first frame of the MD trajectory in GROMACS format.
File Name: stable_open.pdb.
Description: A pdb file of the representative open structure.
File Name: md_fit_dt100ps.xtc
Description: A trajectory file of the stable open state.

Structure, Volume *28*

Supplemental Information

A Refined Open State of the Glycine Receptor Obtained via Molecular Dynamics Simulations

Marc A. Dämgen and Philip C. Biggin

Supplementary Information

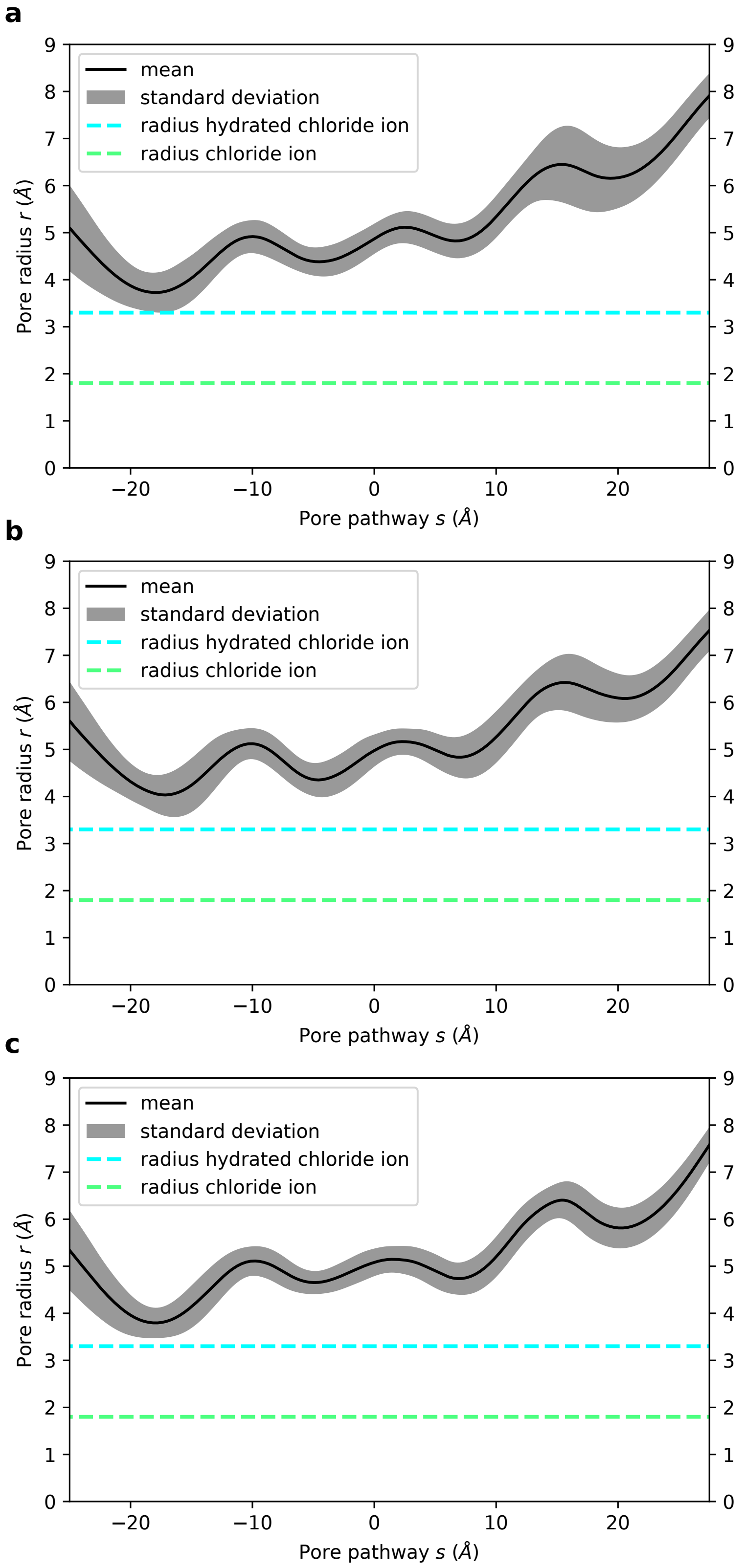

Supplementary Figure 1. Related to Figure 1. Pore profiles from three independent simulations with a stable open state. Time-averaged pore radius profiles of the transmembrane domain obtained from three independent simulations (of 300 ns length each) initiated with different velocities from the system obtained after 150 ns equilibration with pore restraints. No restraints were applied to keep the pore open. The radii of a dehydrated and a hydrated chloride ion are indicated by dashed green and cyan lines, respectively. The pore is positioned in this and subsequent figures such that the L9' ring is located at s = 0. In all three repeats, the structure from the simulation is physically open and theoretically allows permeation of hydrated chloride ions.

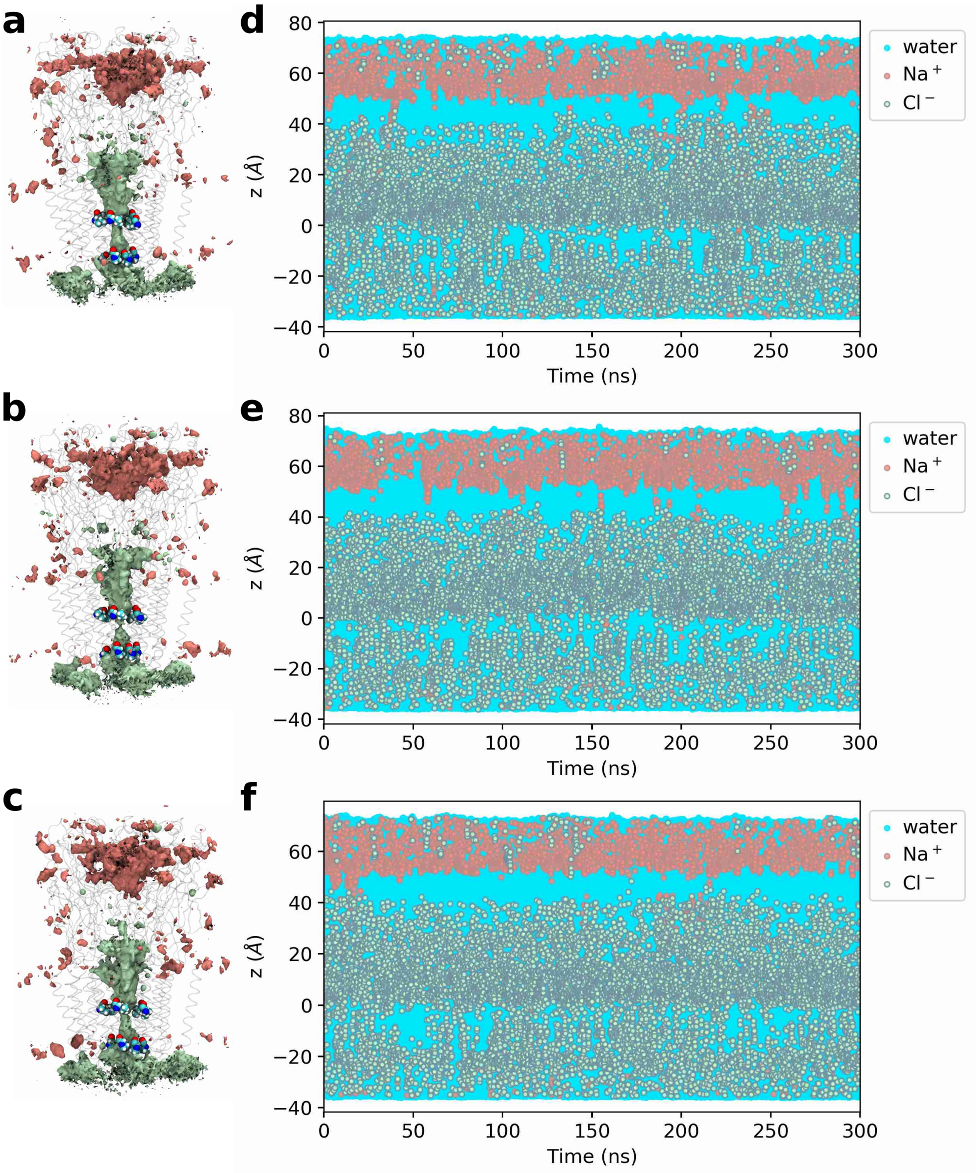

Supplementary Figure 2. Related to Figure 2. Ion densities and trajectories of water and ions within 5 Å of the channel axis for simulations with a stable open state (*150mM NaCl with no membrane potential*). (a-c) Densities of chloride (green) and sodium (red) ions over 300 ns. The isosurfaces represent a density value of 0.5 particles/nm^3. Transparent ribbons indicate the receptor backbone. The L9' ring and P-2' ring residues are shown in van der Waals representation. The densities prove chloride occupancy in the transmembrane pore and, moreover, show selectivity for chloride over sodium in this region. (d-f) Trajectories of water, chloride and sodium ion z-coordinates within 5 Å of the channel axis inside the pore over 300 ns (represented by blue, green and red circles, respectively). The 0 point of the z-axis is positioned at the L9' ring and the receptor structures in the left panels are aligned and scaled correspondingly to allow for spatial orientation along the z-axis. The whole channel pore is wetted throughout the simulations (dewetted regions would appear as white stretches). While chloride frequently penetrates the transmembrane pore, sodium only does so very rarely, again demonstrating the chloride selectivity for this channel.

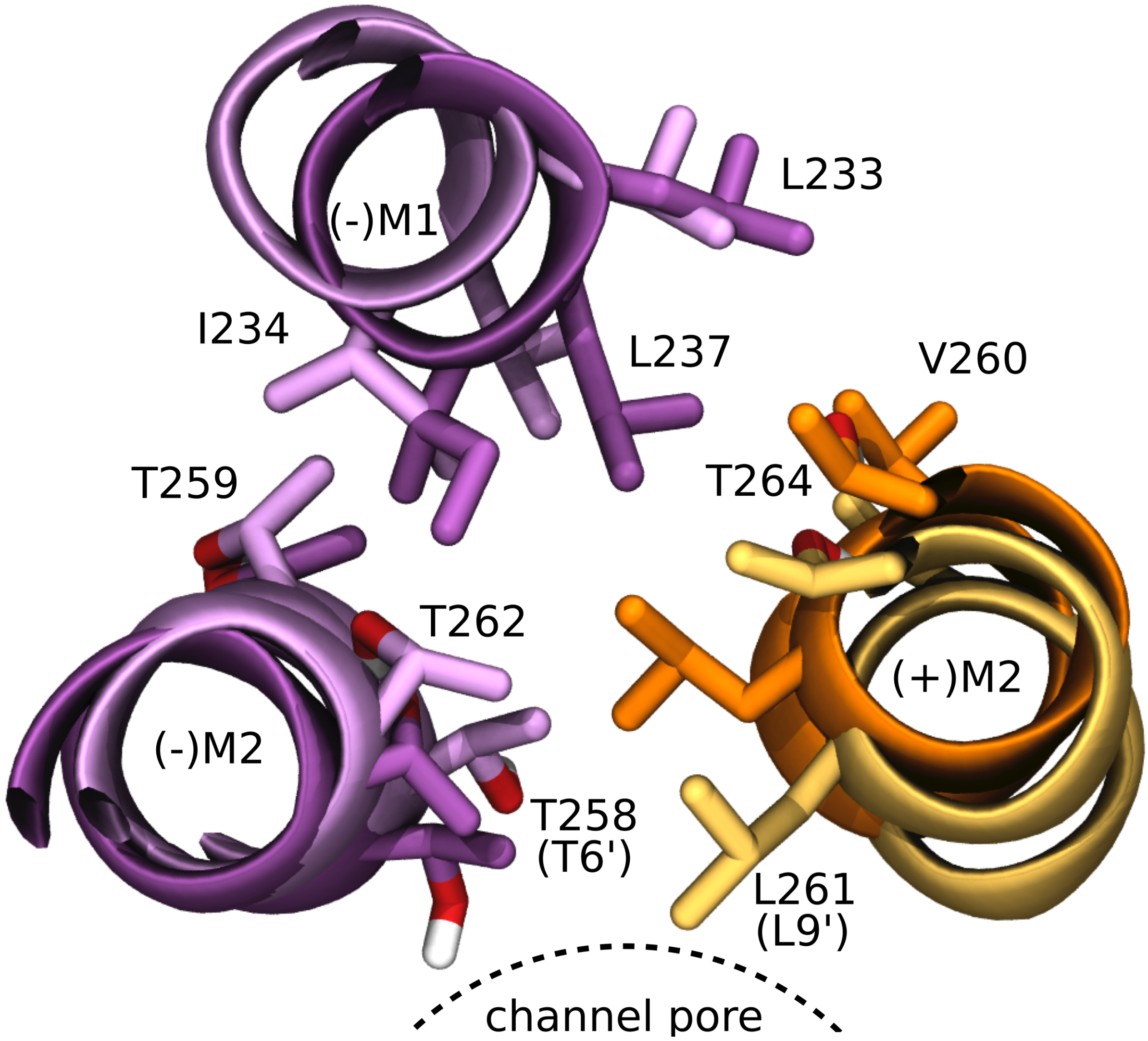

Supplementary Figure 3. Related to Figure 3. Details of the hydrophobic pore and orientation of T6'. Horizontal slice viewed from the extracellular side with the light colours representing the structure at the beginning of the equilibration with pore restraints and dark colours the structure at the end of 150 ns equilibration with pore restraints. The principal subunit is coloured in orange, the complementary subunit in purple. Key residues that form the hydrophobic pocket are indicated. Note also the position of T6' which rotates slightly towards the central pore with its hydroxyl group facing towards the channel, thus facilitating channel hydration.

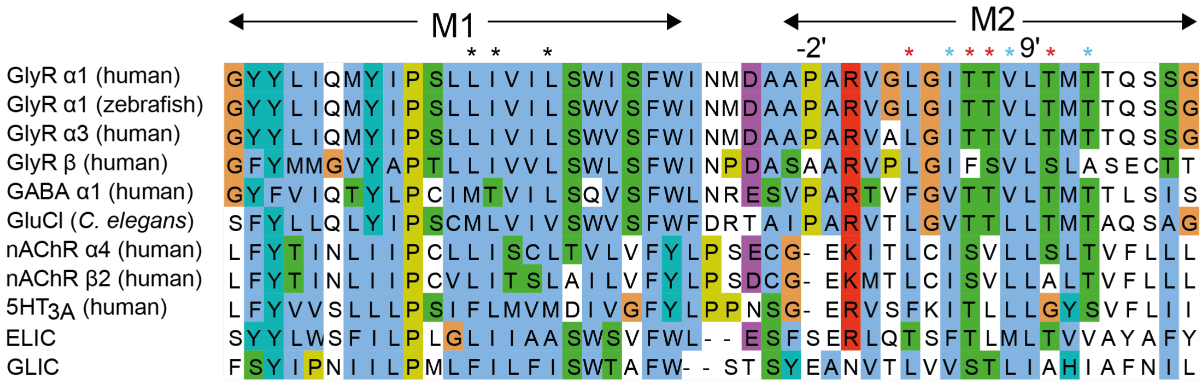

Supplementary Figure 4. Related to Figure 3. Multiple sequence alignment of pLGIC representatives. The conservation of the leucine at L9' is well described. Less well appreciated is the functional conservation of the residues that form the hydrophobic pocket for the leucine in the open state formed by I257, V260 and T264 (I5', V8' and T12' respectively and shown as cyan asterisks) belonging to the M2 helix of the principal (+) subunit, L255, T258, T259, T262 (L3', T6', T7' and T10' respectively and shown as red asterisks) belonging to the M2 helix of the complementary (-) subunit as well as L233, I234 and L237 of the M1 helix of the complementary (-) subunit, shown as black asterisks.

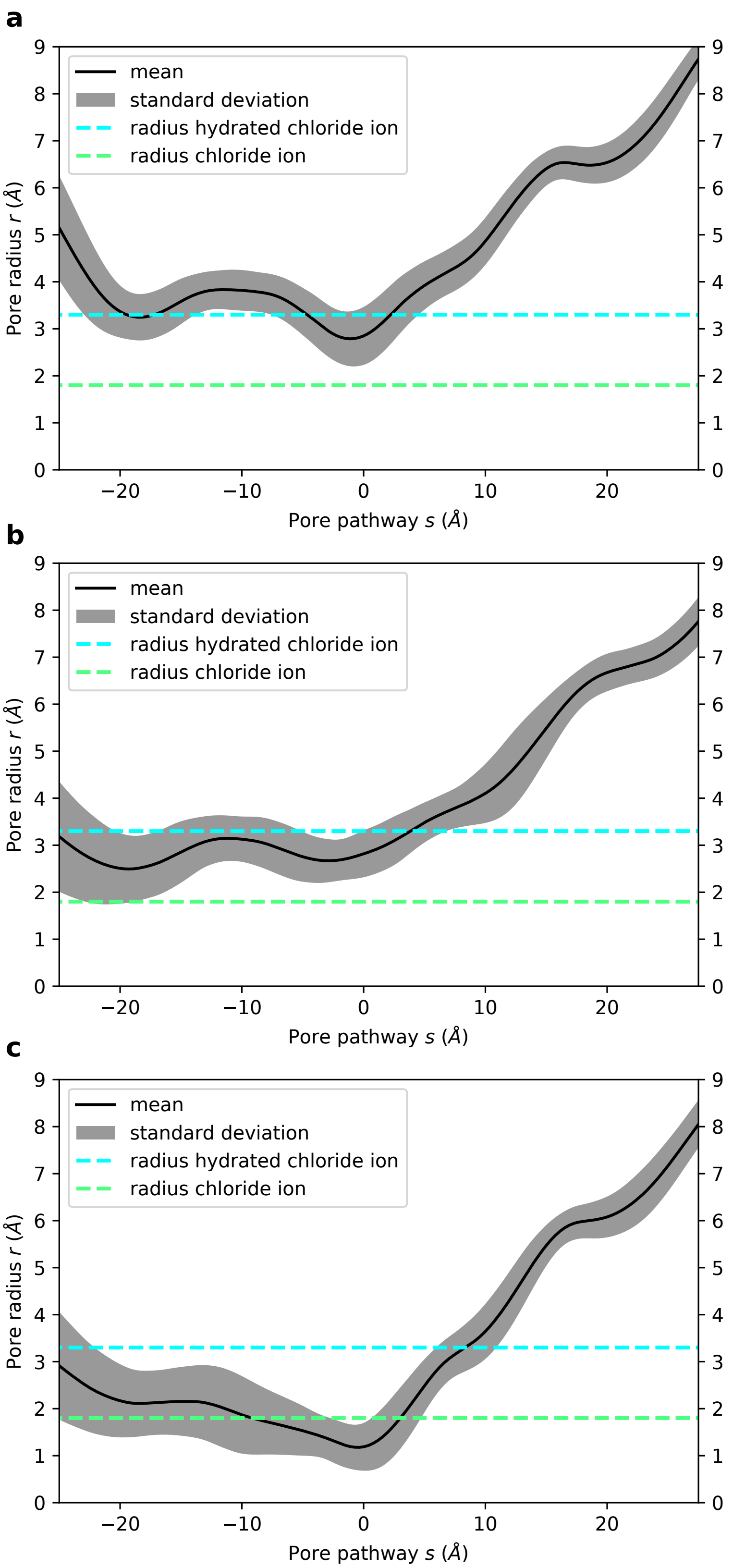

Supplementary Figure 5. Related to Figure 5. Pore profiles from three independent simulations with a collapsed state. Time-averaged pore radius profiles of the transmembrane domain obtained from three independent simulations (of 300 ns length each) initiated with different velocities from the system obtained immediately after the short initial equilibration before the structure is relaxed via the application of minimally invasive pore restraints. The radii of a dehydrated and a hydrated chloride ion are indicated by dashed green and cyan lines, respectively. The L9' and P-2' rings are the main

constriction points. If at all, only partially dehydrated chloride ions could theoretically permeate through the pore.

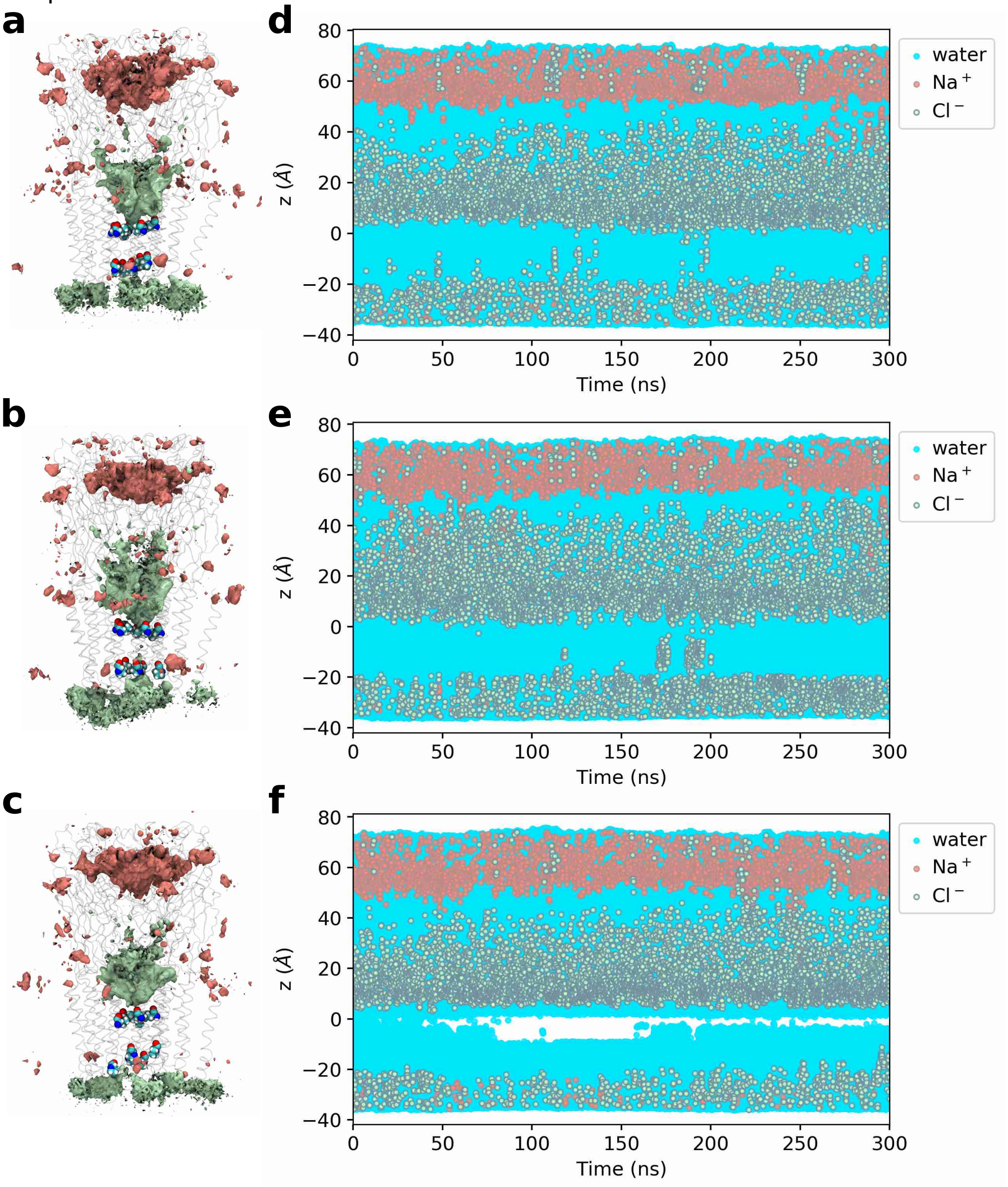

Supplementary Figure 6. Related to Figure 5. Ion densities and trajectories of water and ions within 5 Å of channel axis for simulations with a collapsed state (*150mM NaCl with no membrane potential*). (a-c) Densities of chloride (green) and sodium (red) ions over 300 ns production run. The isosurfaces shown represent a density value of 0.5 particles/nm^3. Transparent ribbons indicate the receptor backbone, The L9' ring and P-2' ring residues are shown in van der Waals representation. The densities show chloride occupancy in the transmembrane pore and moreover, show selectivity for chloride over sodium in this region. (d-f) Trajectories of water as well as chloride and sodium ion z-coordinates within 5 Å of the channel axis inside the pore over 300 ns (represented by blue, green and red circles, respectively). The 0 point of the z-axis is positioned at the L9' ring and the receptor structures in the left panels are aligned and scaled correspondingly to allow for spatial orientation along the z-axis. The white stretches in (c) demonstrate local dewetting of the pore. Chloride ions access the transmembrane pore much less frequently than in the stable open state simulations.

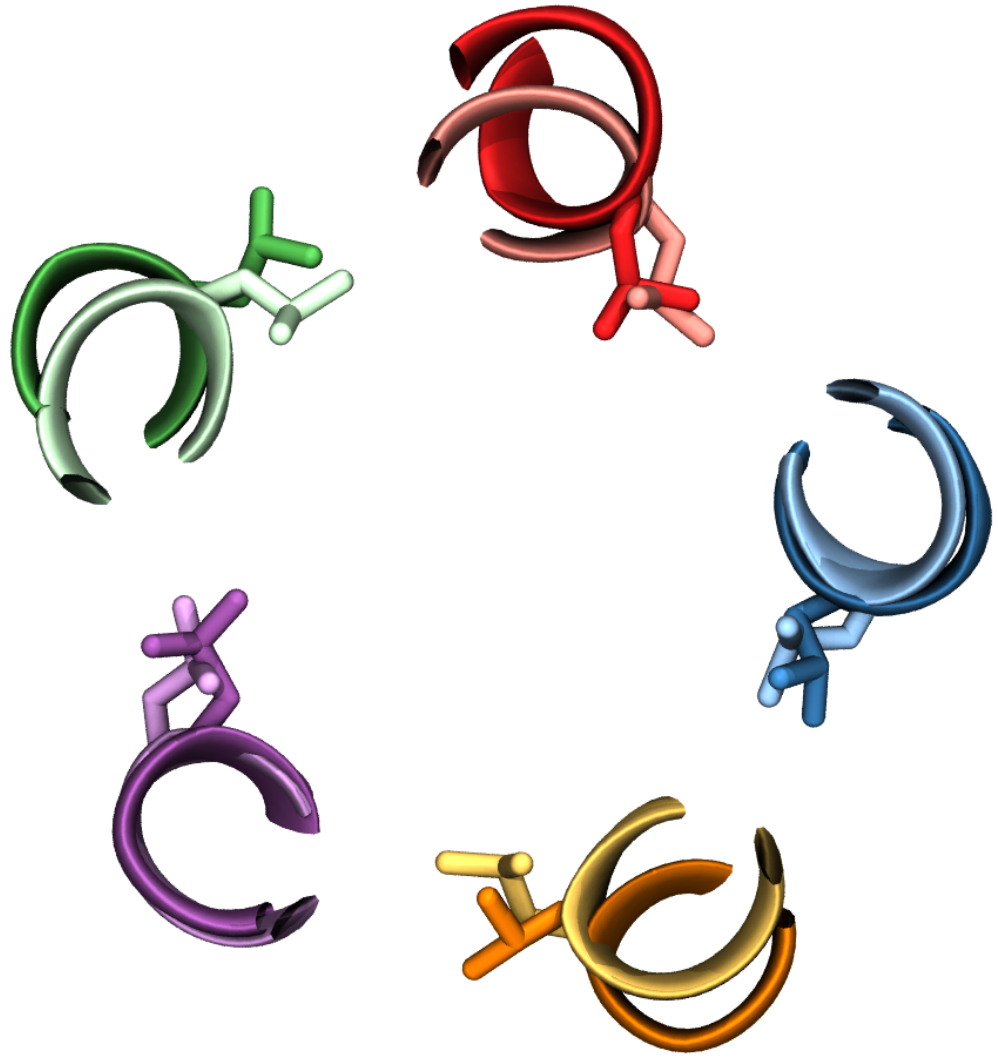

Supplementary Figure 7. Related to Figure 3. Comparison of the leucine gate between the open state glycine and the $5HT_3$ receptor. The open state model (dark colours) was fitted to the Cα atoms of L9' of the $5HT_3$ structure (light colours and PDB code: 6HIN). Shown is a top-down view of a horizontal slice at the L9' position and highlights the similarity in position of the leucines at this position in an open state.

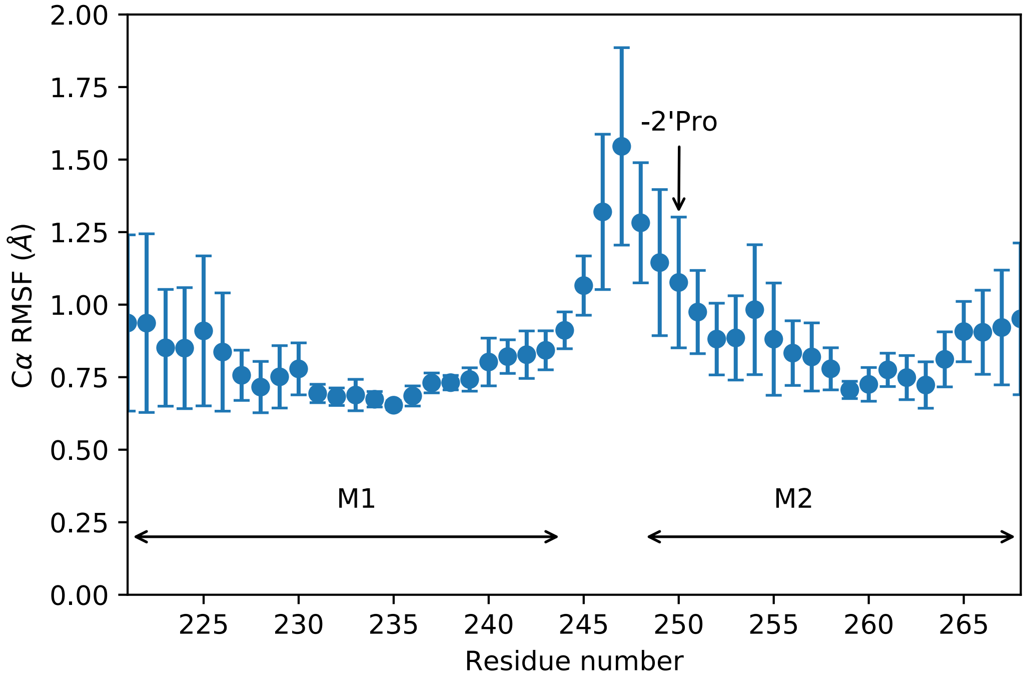

Supplementary Figure 8. Related to Figure 6. Root mean square fluctuation (RMSF) of Cα atoms of M1 and M2 helices. RMSF of Cα atoms of the M1 and M2 helices averaged over all five subunits with standard deviation as error bars. The values corresponding to the region near the intracellular pore opening of the pore lining M2 helix (where the P-2' gate is located) are very high compared to typical values for alpha-helical Cα atoms of around 0.7 Å. This indicates a high conformational flexibility that is accessible at a physiological temperature of 37 °C and can explain the poor density in the cryo-EM map in this region.
